# Integrated vector genomes may contribute to long-term expression in primate liver after AAV administration

**DOI:** 10.1038/s41587-023-01974-7

**Published:** 2023-11-06

**Authors:** Jenny A. Greig, Kelly M. Martins, Camilo Breton, R. Jason Lamontagne, Yanqing Zhu, Zhenning He, John White, Jing-Xu Zhu, Jessica A. Chichester, Qi Zheng, Zhe Zhang, Peter Bell, Lili Wang, James M. Wilson

**Affiliations:** Gene Therapy Program, Department of Medicine, Perelman School of Medicine, University of Pennsylvania, Philadelphia, PA USA

**Keywords:** Genetics research, Translational research

## Abstract

The development of liver-based adeno-associated virus (AAV) gene therapies is facing concerns about limited efficiency and durability of transgene expression. We evaluated nonhuman primates following intravenous dosing of AAV8 and AAVrh10 vectors for over 2 years to better define the mechanism(s) of transduction that affect performance. High transduction of non-immunogenic transgenes was achieved, although expression declined over the first 90 days to reach a lower but stable steady state. More than 10% of hepatocytes contained single nuclear domains of vector DNA that persisted despite the loss of transgene expression. Greater reductions in vector DNA and RNA were observed with immunogenic transgenes. Genomic integration of vector sequences, including complex concatemeric structures, were detected in 1 out of 100 cells at broadly distributed loci that were not in proximity to genes associated with hepatocellular carcinoma. Our studies suggest that AAV-mediated transgene expression in primate hepatocytes occurs in two phases: high but short-lived expression from episomal genomes, followed by much lower but stable expression, likely from integrated vectors.

## Abstract (web summary)

Long-term expression of AAV transgenes in macaque liver is traced to integrated vectors.

## Main

Adeno-associated virus (AAV) gene therapies directed to the liver have been approved for hemophilia A and B, and there are many AAV treatments in late-stage clinical development^1,2^. However, concerns regarding the durability of AAV gene therapies in the liver, along with the challenge of re-administration, have raised questions about its ultimate utility^3–8^. Clinical studies of AAV-based liver gene therapy have demonstrated reductions in efficacy within the first 2 months of treatment^9,10^. Correlations between the appearance of capsid-directed T cells, serum transaminase elevations and reductions of factor IX in hemophilia B trials implicate immune responses in the lack of durability^9^. An immediate reduction in serum bilirubin was observed following AAV gene therapy in an individual with Crigler–Najjar syndrome. However, serum bilirubin returned to pre-treatment levels within 2 months without apparent vector immunity or liver inflammation, suggesting that a non-immune mechanism may lead to loss of efficacy^10^. Following an initial period of expression instability lasting several months, expression appears to be remarkably consistent, albeit at low levels, in both nonhuman primates (NHPs) and humans, which is surprising because the constant turnover of hepatocytes should dilute the episomal AAV genome^11^.

## Results

### AAV gene therapy is efficient but does not persist at high levels in NHP liver

The goal of our study was to define the mechanism(s) that limit efficient and durable transgene expression following liver gene therapy with AAV vectors. Previous studies with complete preclinical and clinical datasets suggest that NHPs are better suited for evaluating key aspects of vector performance than other animal models^9,10,12–16^. We conducted initial studies in rhesus macaques using macaque-derived β-choriogonadotropic hormone (rh-β-CG; *CGB*) as the transgene. rh-β-CG is secreted and has a short serum half-life, meaning it can provide a longitudinal, real-time readout of transgene transcription. As rh-β-CG should be viewed as a self-protein in macaques, confounding adaptive immune responses to the transgene product or to transgene-expressing cells are unlikely. We evaluated two clade E capsids that have been used in multiple clinical trials, AAV8 and AAVrh10 (*n* = 6 NHPs per vector). As an essential and unique aspect of our studies, we analyzed three sequential liver tissue samples from each animal by biopsy at days 14 and 98 and at necropsy at day 182. These analyses included assessment of transgene DNA and RNA by quantitative PCR (qPCR) and cellular distribution of DNA and rh-β-CG protein expression by in situ hybridization (ISH) and immunohistochemistry (IHC), respectively.

**Fig. 1:**
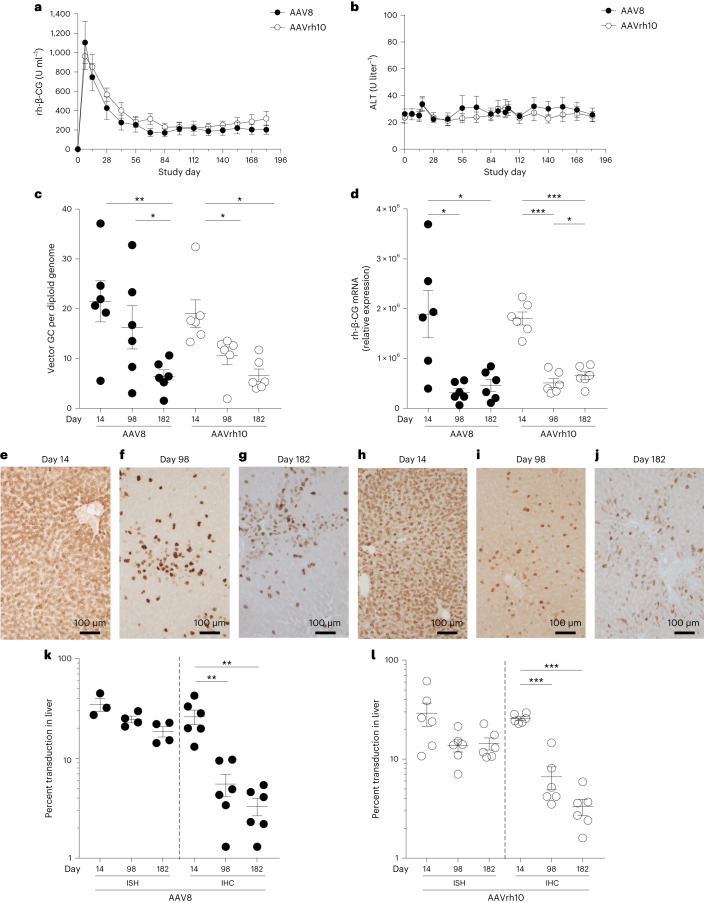
Initial peak followed by a decline to lower stable levels of self-transgene after i.v. administration of AAV vectors to NHPs. **a**,**b**, NHPs received i.v. injections of 10^13^ genome copies per kg (body weight) of AAV8 or AAVrh10 vectors expressing the self-transgene rh-β-CG (*n* = 6 per group). Serum rh-β-CG levels were evaluated throughout the in-life phase for transgene expression (**a**) as well as alanine aminotransferase (ALT) levels (**b**). Liver tissue was collected during a liver biopsy procedure (14 or 98 days after vector administration) or at the time of necropsy (182 days after vector administration). **c**,**d**, DNA (**c**) and RNA (**d**) were extracted from liver samples to evaluate the number of vector genome copies (GC) and transgene RNA levels, respectively. **e**–**j**, IHC for rh-β-CG protein was performed on liver samples (brown staining) from animals administered AAV8 (**e**–**g**) or AAVrh10 (**h**–**j**). ISH was performed on liver samples using the RNAscope Multiplex Assay. Hybridized probes were imaged with a fluorescence microscope. **k**,**l**, Vector DNA was quantified by ISH, and transgene expression was determined by IHC as the percentage of AAV^+^ cells for NHPs administered AAV8 (**k**) or AAVrh10 (**l**). Values are presented as mean ± s.e.m.; *P < 0.05; **P < 0.01; ***P < 0.001. Source data

We observed similar levels and profiles of rh-β-CG protein expression in serum for each capsid; peak levels were achieved by day 7, followed by a gradual decline to stable levels three- to sixfold lower than the peak (Fig. 1a) without transaminase elevations (Fig. 1b). Statistically significant reductions in total vector DNA and RNA occurred over time, although the magnitude of reduction was smaller for DNA than for RNA. Although DNA levels decreased further over the two later time points, RNA levels were stable between days 98 and 182 after the initial decline relative to day 14 (Fig. 1c,d).

To elucidate the mechanism governing this rapid decline in expression, we evaluated the cellular distribution of rh-β-CG protein expression by IHC (Fig. 1e–j and Supplementary Fig. 1) and nuclear DNA by ISH using a probe specific for vector DNA as part of a probe pair to target DNA and RNA separately (Fig. 1k,l). The number of rh-β-CG^+^ cells declined three- to fivefold from day 14 to day 98 and then remained relatively stable through day 182, consistent with the kinetics of rh-β-CG in serum and transgene RNA expression in the liver. The approximately fourfold reduction in rh-β-CG-expressing cells was not associated with a commensurate reduction in DNA-containing cells, which decreased by only 24–53%.

### Vector DNA assembles into discrete nuclear domains that persist despite the loss of transgene expression

We next evaluated the same parameters of gene transfer and expression in macaques intravenously (i.v.) administered AAV8 vectors expressing one of three transgenes: a reporter gene encoding green fluorescent protein (GFP) or a transgene encoding the human or macaque version of the low-density lipoprotein receptor (hLDLR and rhLDLR, respectively; *n* = 2 per transgene). These studies allowed us to assess the role of adaptive immunity in the efficiency and stability of transgene expression within a range comprising the highly immunogenic protein GFP to the non-immunogenic self-protein rhLDLR.

**Fig. 2:**
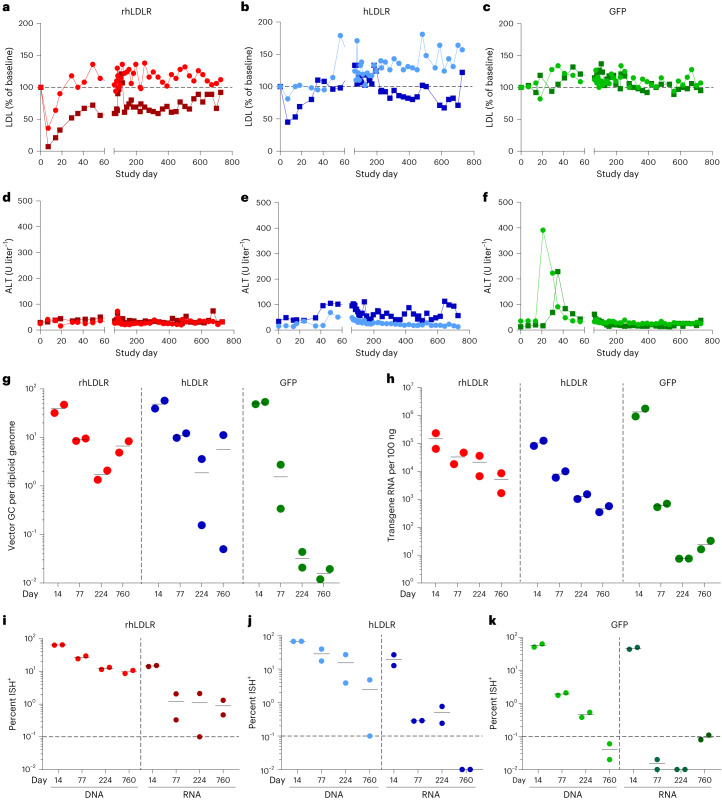
Comparison of the level, duration and localization of expression of self, human and non-self transgenes following i.v. administration of AAV vectors. **a**–**f**, NHPs received i.v. injections of 10^13^ genome copies per kg (body weight) AAV8 vectors encoding rhLDLR, hLDLR or GFP (*n* = 2 per group). Serum LDL (**a**–**c**) and ALT (**d**–**f**) levels were evaluated throughout the in-life phase. Liver tissue was collected during a liver biopsy (14, 77, and 224 days after vector administration) or necropsy (760 days after vector administration). **g**,**h**, DNA (**g**) and RNA (**h**) were extracted from liver samples to evaluate the number of vector genome copies and transgene RNA levels, respectively. **i**–**k**, ISH was performed on liver samples using the RNAscope Multiplex Assay. The probes used were non-overlapping probe pairs, in which one probe was specific for DNA (binding to the antisense strand), and the second probe hybridized to RNA. Hybridized probes were imaged with a fluorescence microscope. The vector DNA and transgene RNA were quantified as the percentage of AAV^+^ cells for animals treated with AAV vectors expressing rhLDLR (**i**), hLDLR (**j**) or GFP (**k**). Light and dark colors indicate each individual NHP in the cohort; red (rhLDLR), blue (hLDLR) and green (GFP). The dashed lines indicate the level where background can be observed due to autofluorescence. Source data

Throughout the in-life phase of the study, we used serum LDL levels as an indirect assessment of transgene expression that should reflect the levels of transgene-derived LDLR in real time (Fig. 2a–c). Animals that received the rhLDLR vector showed an acute and substantial reduction in serum LDL, which returned to levels close to or at baseline within 30 days (Fig. 2a). We observed a similar pattern of serum LDL for the hLDLR vector but with a lower magnitude of transient reduction (Fig. 2b). As expected, we did not observe a reduction in serum LDL for the GFP vector (Fig. 2c). The reduction in serum LDL as a result of transient LDLR expression was associated with an expected acute but transient reduction in serum PCSK9 (Supplementary Fig. 2). Serum transaminase levels tracked with the expected immunogenicity of the transgene, ranging from no elevations with rhLDLR (Fig. 2d) to mild elevations with hLDLR (Fig. 2e) to a sharp and transient increase with GFP (Fig. 2f). Sequential measurements of T cells by enzyme-linked immunosorbent spot revealed transgene responses in five of six animals, with the extent of transaminase elevation being commensurate with the degree of transgene immunogenicity (GFP > hLDLR > rhLDLR); T cell activation to the capsid was minimal (Supplementary Fig. 3).

We performed tissue studies as described for the rh-β-CG experiments, although four samples were available for each animal spanning a longer time frame (that is, biopsies at days 14, 77 and 224 and necropsy at day 760). Analyses of vector DNA and transgene RNA (as measured by qPCR) followed the same trends over time as those for rh-β-CG across all transgenes, showing reductions in both DNA (Fig. 2g) and RNA (Fig. 2h), with greater losses in RNA than in DNA. The extent of these reductions was much greater for the transgene encoding GFP (DNA diminished >3,000-fold, and RNA diminished >55,000-fold) than for the transgene encoding rhLDLR (DNA diminished 6-fold, and RNA diminished 29-fold). The hLDLR vector results were more similar to the data for the rhLDLR vector, showing DNA and RNA reductions of 9-fold and 227-fold, respectively.

We performed ISH to characterize the number of cells harboring intranuclear vector DNA compared to those with cytoplasmic transgene-derived RNA (Supplementary Fig. 4). The pattern observed for the rhLDLR vector was similar to the rh-β-CG vector results, with high numbers of DNA-containing and RNA-expressing cells at day 14 (65% and 15%, respectively), followed by a 7-fold reduction of DNA over 2 years and a 12-fold reduction in RNA-expressing cells by day 77. The number of RNA-expressing cells then remained stable through day 760, reaching a steady state of ~1% RNA-expressing cells (Fig. 2i and Supplementary Fig. 4). The pattern for hLDLR was essentially the same, although with greater reductions in DNA (28-fold) and RNA (~3,000-fold), with RNA-expressing cells falling below the 0.1% threshold of detection at day 760 (Fig. 2j and Supplementary Fig. 4). Animals that received the vector encoding GFP exhibited the same high level of gene transfer and transgene expression based on analyses for day 14, although the expression dropped to undetectable levels by day 760 for DNA and by day 77 for RNA (Fig. 2k and Supplementary Fig. 4).

**Fig. 3:**
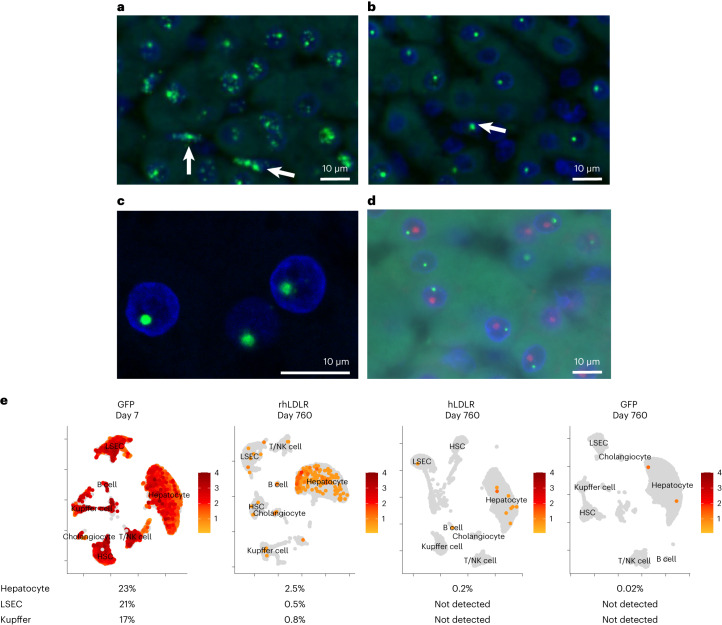
Cell type and lack of substructure association of vector DNA and RNA following i.v. administration of AAV vectors to NHPs. **a–e**, NHPs received i.v. injections of 10^13^ genome copies per kg (body weight) of AAV8 vectors encoding rhLDLR, hLDLR, or GFP (*n* = 2 per group). Liver tissue was collected during a liver biopsy (14 days after vector administration) or necropsy (760 days after vector administration). ISH was performed on liver samples using a DNA-specific probe (binding to the antisense strand). **a**,**b**, DNA ISH images from NHPs administered rhLDLR at days 14 (**a**) and 760 (**b**) after vector administration. Arrows mark cells with DNA ISH signal that are not hepatocytes; green, vector DNA; blue, DAPI (nuclear counterstain). **c**,**d**, Hybridized probes were imaged with a confocal microscope (**c**) and co-stained with a nucleolus marker (fibrillarin antibody) shown in red (**d**). **e**, Nuclei were extracted from necropsy samples (day 760), and cDNA libraries were created from single nuclei. Nuclei from similar cell types cluster together, and the total percentage of nuclei expressing transgene RNA was evaluated. To enable analysis of transduction at an early time point, we used samples from two additional animals previously treated with 7.5 × 10^12^ genome copies per kg (body weight) of AAV8.TBG.GFP and necropsied at day 7 (ref. ^17^). A representative uniform manifold approximation and projection (UMAP) is shown for each group; NK cell, natural killer cell; HSC, hepatic stellate cell. Source data

DNA-specific ISH revealed two patterns of intranuclear hybridization at day 14: a diffuse granular pattern and a single bright circular structure (Fig. 3a). Evaluations at later time points demonstrated retention of the circular structures, with a loss of the background granular staining (Fig. 3b). Confocal imaging illustrated the spherical shape of these structures with a diameter between 0.8 and 1.6 µm (Fig. 3c). We co-stained sections for vector DNA by ISH and for fibrillarin as a nucleolus marker by IHC. There was no overlap between these two structures, indicating that the single nuclear domain that harbors vector genomes is not related to the nucleolus (Fig. 3d).

To better understand the global picture of liver transduction with respect to cell type, we performed single-nucleus RNA sequencing (snRNA-seq; Fig. 3e and Supplementary Fig. 5). To enable analysis of transduction at an early time point, we used samples from two additional animals previously administered AAV8.TBG.GFP and necropsied at day 7 (ref. ^17^). snRNA-seq analysis of these liver tissues at day 7 revealed an ~20% transduction of multiple cell types, including hepatocytes, liver sinusoidal endothelial cells (LSECs) and Kupffer cells, indicating that the thyroxine-binding globulin (*TBG*) promoter is not hepatocyte specific. The same analysis of day 760 tissues (Fig. 3e and Supplementary Fig. 5) showed reduced transduction similar to that for other measurements of transgene expression in hepatocytes (that is, RNA PCR and RNA ISH), with transduction of 2.5% for rhLDLR, 0.2% for hLDLR and 0.02% for GFP (Fig. 3e). Only rhLDLR-treated animals had significant detectable transgene expression in non-hepatocytes (that is, LSECs and Kupffer cells) at day 760.

The DNA ISH analyses from rhLDLR tissues were re-evaluated for evidence of vector genomes in non-parenchymal cells in light of the snRNA-seq results that showed expression across multiple cell types. Nuclear staining was indeed demonstrated at day 14 time points in non-hepatocytes, although this was substantially reduced by day 77 (Fig. 3a,b). The relevance of the wide distribution and expression of the AAV genome throughout different liver cells is unclear to the issue of durability being studied in this manuscript, although it could be important in understanding toxicity of systemic AAVs.

### Vector integrates at high frequency throughout chromosomal DNA as complex concatemeric structures

The presence of many non-expressing hepatocytes that continued to harbor vector DNA compelled us to more fully characterize the structure and location of the vector genomes detected by ISH. We and others have shown integration of AAV genome sequences into chromosomal DNA in the setting of DNA repair following double-stranded breaks^18–25^. We evaluated liver DNA from rh-β-CG- and LDLR/GFP-treated animals for integrated vector sequences using inverted terminal repeat sequencing (ITR-seq), an anchored multiplexed PCR-based next-generation sequencing method developed by our group to detect AAV ITR sequences that have integrated into the genome^18^. This method captures the chromosomal sequences directly adjacent to insertion sites by ligating specific unique molecular identifier (UMI)-containing adapters to sheared DNA. This is followed by PCR amplification using primers specific to the AAV ITR and ligated adapter sequences. AAV integration sites are determined from chimeric sequencing reads that contain both AAV ITR DNA and the adjacent host chromosomal DNA. The numbers and locations of unique genome–AAV junctions were determined for each sample, and the adjacent genomic DNA sequences in each unique AAV integration were further characterized and annotated.

**Fig. 4:**
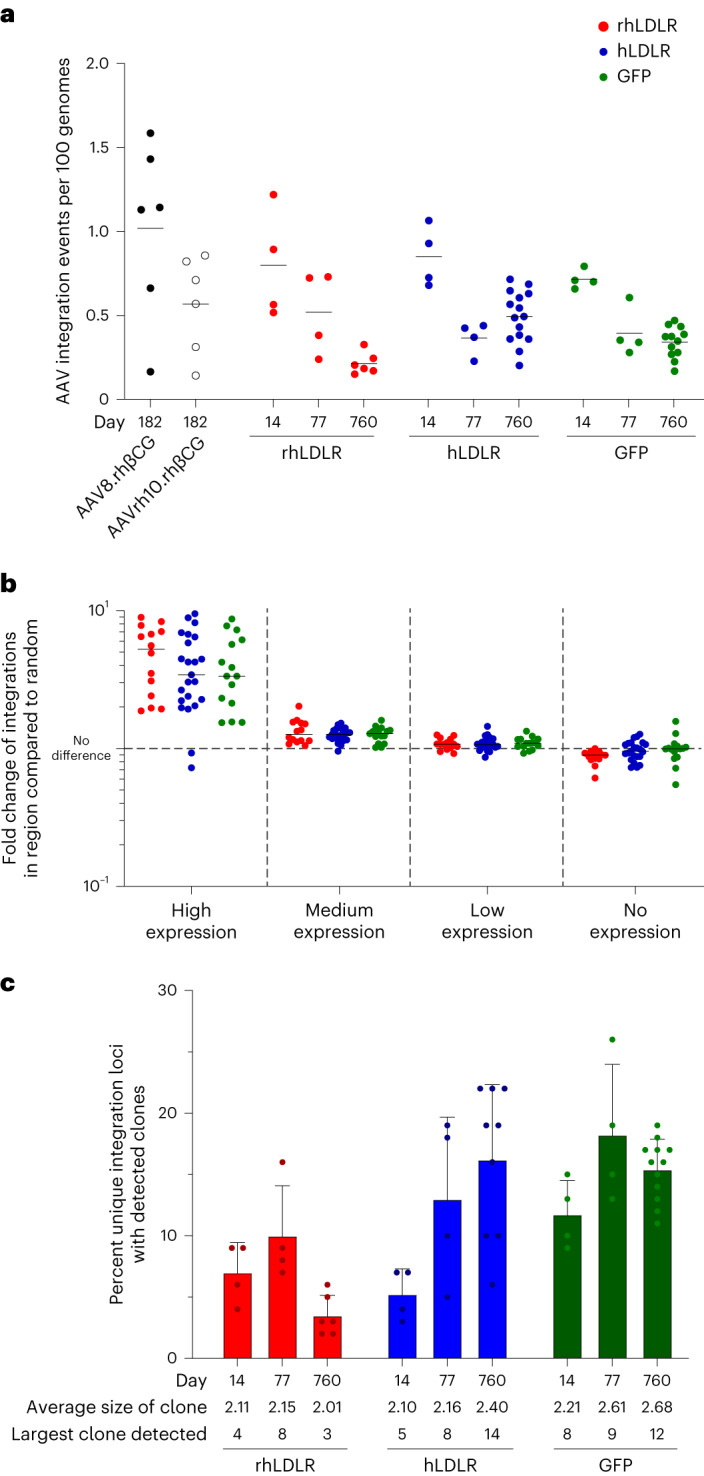
Localization of integrated vector DNA following i.v. administration of AAV vectors to NHPs. **a**–**c**, NHPs received i.v. injections of 10^13^ genome copies per kg (body weight) AAV8 vectors encoding rhLDLR, hLDLR or GFP (*n* = 2 per group). Liver tissue was collected during a liver biopsy procedure (14, 77 and 224 days after vector administration) or at the time of necropsy (760 days after vector administration). **a**, DNA was extracted from liver samples, and the number of AAV integration loci in all injected NHPs was determined by ITR-seq. The number of unique integration loci was normalized to 100 genomes based on input DNA. Each NHP had two samples run at each time point, except for at day 760 when several replicates of each NHP were performed. **b**, The genomic locations of AAV integration loci were determined by ITR-seq and annotated as being within a gene-coding region (genic) or outside of a gene-coding region (intergenic) according to the rhesus macaque RefSeqGene annotation. Integrations within genic regions were further annotated by the distribution of RNA expression in the human liver (data were taken from the Human Genome Atlas at www.proteinatlas.org). Expression levels were determined by normalized expression (nx) in the human liver for each annotated gene. The following categories were determined: genes not expressed in liver (1 < nx), genes with low expression in liver (1 ≥ nx < 10), genes with medium expression in liver (10 < nx > 100) and genes with high expression in liver (100 ≥ nx). A random distribution represents 10,000 randomly computationally generated genomic loci in the rhesus macaque genome. The number of AAV integration events in each gene category is presented as a fold change over random sequences. A value of 1 would represent no difference from the distribution of random loci (dashed line). Data are presented as mean ± s.e.m. **c**, The numbers of clones for each unique integration loci detected were determined by requiring the same insertion site and different adapter positions and different UMIs for each clone. The percentage of unique insertion sites that were clonally expanded (two or more clones) is represented in the graphs. Samples were the same as assessed in **a**. Of clonally expanded loci, the average number of clones and the largest clone detected between the two NHPs for each group is listed below the graph. Data presented as mean ± s.e.m. Source data

Analysis of tissues at day 182 from rh-β-CG vector-treated animals showed integration events at frequencies of 1.6 per 100 genomes to 1 per 1,000 genomes (Fig. 4a). A time course of integrations performed in the GFP/LDLR study showed similar levels of integration events, which declined between days 14 and 77 and subsequently stabilized to levels of 0.1–0.7 AAV integration events per 100 genomes (Fig. 4a). These integrations occurred across the genome and mostly followed a widely dispersed distribution pattern, with the exception of an increase in integrations in and around genes that are highly expressed in the liver (Fig. 4b and Supplementary Fig. 6). None of the identified insertion sites from any of the evaluated NHP samples were located within genic regions frequently mutated in human hepatocellular carcinomas (HCCs; including *TP53*, *TERT*, *CTNNB1* and so on^26–28^) nor in genic regions identified in the development of mouse HCC following AAV treatment (*Dlk1*, *Tax1bp1*, *Meg8* (the mammalian ortholog of the Rian locus) and so on)^29–32^.

To determine whether any of the detected insertion sites exhibited evidence of clonal expansion, we used the UMIs in the ITR-seq reads and the exact nucleotide position of the genomic DNA adjacent to the adapter to remove PCR duplicates and identify and quantify clonal expansion of unique insertion sites, similar to the technique described by Nguyen et al.^33^ We defined clonal expansion as two or more unique integrations (as determined by independent UMI sequences and adapter locations) at the same genomic location. Samples were evaluated for the percentage of integrations with clonal expansion, the average size of detected clones and the largest detected clone within each sample (Fig. 4c). Animals treated with GFP and hLDLR exhibited an increased number of expanded loci and a larger overall size of clones than rhLDLR-treated animals. For all three groups, the largest increase in clonal integration loci was between days 14 and 77. The size of the largest clone in each sample was similar to results of a recent study with AAV-transduced human hepatocytes expanded in a mouse model of xenogenic liver regeneration^34^, both of which are relatively low compared to observations in AAV-treated dogs up to 10 years after vector administration, where vector-induced expansion was proposed^33^.

To fully characterize the composition of AAV sequences at late time points after vector administration, we used multiple long-read single-molecule sequencing techniques. We first performed long-read sequencing of DNA from vector preparations used for animal dosing to assess the integrity of administered AAV genomes. This approach showed complete ITR-to-ITR vector genomes represented the majority of AAV DNA; however, this approach also revealed a minority population of genomes with truncations that were not present in the input plasmid (Supplementary Fig. 7a). To determine whether a similar proportion of AAV DNA remained intact and was able to drive transgene expression in vivo, we proceeded to also characterize in vivo vector genomes by long-read sequencing. Due to the relatively low abundance of AAV DNA in vivo and its existence as both episomes and broadly distributed integrants, we used a hybridization-based enrichment approach based on binding to biotinylated oligonucleotides tiled across the individual transcriptional units to enrich for vector-containing DNA sequences. The assay was performed with necropsy samples collected at day 760, when adequate material was available for the pulldown enrichments. Sequencing of enriched DNA was performed using high-fidelity (HiFi) circular consensus sequencing (CCS) on a PacBio Sequel II instrument (>99% accuracy and *Q* > 20). The CCS reads were then analyzed for the presence of vector and host genome sequences. This approach yielded individual reads ranging from 51 to 50,419 base pairs (bp) in length, with average sequence sizes ranging from 4,216 to 6,113 bp. The number of reads that contained vector sequences was proportional to the total amount of vector DNA originally detected by qPCR, with rhLDLR > hLDLR > GFP (Table 1).

**Table 1:** Summary of liver-directed gene therapy outcomes at 2 years after i.v. administration of AAV vectors to NHPs

	GFP1	GFP2	hLDLR1	hLDLR2	rhLDLR1	rhLDLR2
ISH RNA (%)	0.08	0.11	0.01	0.01	0.5	1.3
10x + RNA (%)	0.01	0.02	0.29	0.05	1.0	2.7
ISH DNA (%)	0.02	0.06	4.8	0.1	11	9
DNA (genome copies per cell)	0.02	0.01	11	0.1	5	8
Short-read sequencing, number of unique integration sites per 100 cells	0.7	0.4	0.6	1.8	0.8	0.6
Long-read sequencing						
Total	0.9 M	1.5 M	2.1 M	4.2 M	2.6 M	2.6 M
Vector	391	201	1,457	504	7,394	6,907
Vector + flank (confirmed integrated)	266	2	30	58	1102	939
Average no. ITRs per read	2.52	1	1.57	2.9	3.32	3.11
Functional cDNA	0	52	24	167	3,269	2,653
cDNA + flank (confirmed integrated)	0	0	0	21	426	314

ISH for DNA and RNA (Fig. 2i–k), snRNA-seq (Supplementary Fig. 5) and vector DNA in total genomic DNA (Fig. 2g). Integration per 100 cells is the number detected by ITR-seq (Fig. 4a), with values adjusted based on long-read sequencing results. High-molecular-weight DNA was also extracted from liver samples and was enriched by hybridization to probes that tiled the vector sequence for HiFi long-read sequencing by PacBio. Total represents the total number of CCS HiFi reads per sample; M, million. Vector represents the total reads with ITR-to-ITR vector genome sequence. Vector + flank represents total reads with ITR-to-ITR vector genome sequence and flanking host genome sequence. Average ITR per read represents the average number of ITRs present within a vector + flank read. cDNA represents the total reads with at least one functional copy of the transgene. Functional promoter directly upstream represents the total reads with functional cDNA that have a promoter sequence directly upstream. cDNA + flank represents total reads with at least one functional copy of the transgene and flanking host genome sequence.

**Fig. 5:**
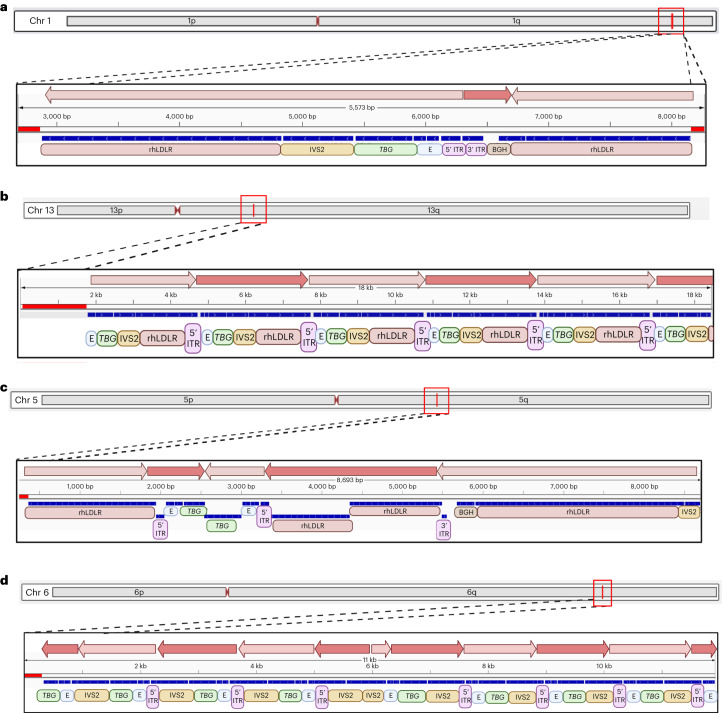
Structure of integrated vector DNA following i.v. administration of AAV vectors to NHPs. **a**–**d**, NHPs received i.v. injections of 10^13^ genome copies per kg (body weight) of AAV8 vectors encoding rhLDLR, hLDLR or GFP (*n* = 2 per group). Liver tissue was collected at necropsy (760 days after vector administration) for each NHP, and high-molecular-weight DNA was extracted from liver samples and enriched by hybridization to probes that tiled the vector sequence. HiFi long-read sequencing of liver DNA was performed using high consensus accuracy CCS on a PacBio Sequel II instrument (>99% accuracy and *Q* > 20). CCS reads were mapped to both the vector and host genomes. For CCS reads containing host genomic sequence, the host genomic sequence is indicated below the scale bars as red boxes and the location of integrated vector DNA was determined as indicated by the red lines marked on each chromosomal picture. Blue boxes indicate vector genome sequences, which were further annotated by component (5' ITR; enhancer, E; promoter, *TBG*; intron, IVS2; transgene, rhLDLR; PolyA, BGH; 3' ITR) and by orientation (arrows above scale bar). **a**, A complete integration would contain two chromosomal junctions flanking the entire integration. **b**–**d**, Most integrations were complex concatemers and were incompletely captured, as represented by only one chromosomal junction. Vector integrations in head-to-tail (**b**), head-to-tail and head-to-head (**c**) and head-to-tail and tail-to-tail (**d**) complex concatemeric configurations were seen.

Sequence-based analysis of these vector-containing reads demonstrated remarkable heterogeneity in the structure of AAV sequences present in vivo (Fig. 5), which often were present in complex concatemers containing mixtures of rearranged and truncated genomes (examples of these complex concatemers are presented in Fig. 5a–d). Mapping the reads against the input vector sequence illustrated the extensive nature of the rearrangements/truncations that could not have been explained by erroneous input vector and instead must have occurred after administration (compare Supplementary Fig. 7a and 7b–d).

To help determine the likelihood that these rearranged genomes retained the ability to express transgene, we evaluated the individual long-read sequences for the presence of intact transgene cDNA. The number of intact cDNA sequences was very low in the GFP group and increased in the LDLR groups, with rhLDLR > hLDLR; in each group, the number of reads with cDNA sequences was 0-44% of those that showed any vector sequence.

A subset of cDNA containing sequences (that is, ~12%) were shown to contain flanking rhesus macaque genomic sequence in at least one end, suggesting that they were derived from integrated genomes. By simultaneously quantifying the presence of genomic and AAV DNA, we estimated that for rhLDLR samples (where the most integration was seen), between 33% and 39% of integrated sequences contained a fully intact transgene cDNA. However, this likely represents an underestimate because of shearing of the DNA during isolation and premature termination of the sequencing run.

Of all reads containing at least one flanking sequence, 92.9% contained at least one ITR, and 85.2% contained two or more ITRs. Only 10.2% of the ITRs were intact, with the majority showing heterogeneous truncations near a common region of the B–B′ palindromic loop, resulting in an average ITR length of 102.6 bp (Supplementary Fig. 8)^35,36^. There was remarkable homogeneity in ITR sequences within reads that harbored more than one ITR and substantial variation between ITR sequences of different reads. We broadly assessed variations within the genomic flanking DNA to provide insight into the consequences of integration on genome integrity. Based on all reads containing at least one flanking sequence, the genomic DNA was largely intact (96.8%), with only 0.7% insertions and 2.6% deletions in the flanking genomic DNA. We captured at least four long reads with flanking genomic sequence on both ends of a vector sequence. In these examples, flanking host genomic sequences demonstrated a low percentage of mismatches (94–100% identity); in a few cases, some of the flanking sequences were flipped in orientation and/or repeated, but otherwise, the rhesus macaque genome was largely unperturbed.

## Discussion

Our studies reveal a complex interplay between adaptive immunity and DNA repair that affects the performance of AAV gene therapy in the primate liver. An important finding was the formation of large discrete nuclear domains of vector DNA that persist despite concomitant reductions in transgene expression. The presence of these nuclear domains is reminiscent of the replication centers observed during viral infection with adenovirus and herpes simplex virus. One hypothesis is that transcriptionally active genomes located within these structures, or elsewhere in the nucleus, undergo epigenetic silencing in a similar manner to the inactivation of intranuclear genomes of viruses (such as herpesviruses) by innate host responses. Another potential explanation is that genomes that are transcriptionally active soon after delivery are unstable^37^, such as the transiently appearing vector DNA that is widely dispersed in the nucleus, while the vector genomes within the persisting discrete nuclear domains were never transcriptionally active. Transgene-specific biology (for example, circulating half-life, transgene activity and biodistribution) could also contribute to variability in the pattern of expression observed for several AAV gene therapies over time^9,16^. The persistence of these non-expressing nuclear vector domains helps explain the long-standing enigma in primate liver gene therapy studies of high vector DNA in the setting of low vector RNA^38,39^.

The demonstration of integrated vector sequences at levels of 1 per 100 cells informs the consideration of safety and efficacy of liver gene therapy in several ways. The abundant and broad distribution of integrations has the potential to lead to malignant transformation. However, none of these integrations were within genes known to be associated with HCC, nor was there evidence of marked clonal expansion during the 2 years of follow-up. The fact that integration events are far less common (about 1%) than the number of cells that express at day 14 (that is, ~20%) and the number of cells that retain vector DNA within the nuclear domain (that is, ~10%) indicates that episomal forms of the vector genome must be responsible for early transcription and the formation of discrete nuclear domains. Similarities in the frequency of integration sites to the number of long-term expressing cells as measured by ISH/IHC and snRNA-seq (~1%) suggests that integration may be responsible for durable expression (summarized in Table 1). Long-read sequencing revealed the presence of vector genomes in large complex concatemers harboring many truncations and rearrangements. Evidence to support integration of these concatemers was provided in a subset of reads that contained flanking macaque chromosomal DNA. Limitations in the capture of complete concatemeric structures by long-read sequencing precluded a quantitative assessment of integration frequency, although the presence of integrated sequences with intact vector genomes supports the hypothesis that integrated vectors may be responsible for long-term expression. The finding of high vector DNA in the setting of low vector RNA was shown in our studies with two capsids and three transgenes and was similar to that described by others using different promoters, suggesting that this is a class effect of AAV in primate liver^38,39^. However, we cannot rule out attributes of the vector, such as promoter sequence, in influencing expression profiles.

Our studies also demonstrate the impact that adaptive immune responses can have on the frequency and molecular state of the vector DNA (for example, episomal versus integrated DNA). In the case of a non-immunogenic transgene, ~10 genome copies per cell are distributed across nuclear domains present in ~10% of cells, with unique integrations detectable in ~1 out of 100 cells at later time points. Activation of a T cell response to the transgene as observed with GFP substantially reduced the overall vector DNA content to 0.02 genome copies per cell and eliminated most of the nuclear domains to only 0.04% of cells without impacting the number of detected unique integration sites (~1 integration locus per 100 cells). Immune-mediated elimination of episomal viral DNA genomes is a well-known host defense mechanism^40–42^. It is interesting that animals treated with immunogenic transgenes demonstrated more truncations/rearrangements, thereby suggesting that cells expressing functional vector genomes are eliminated over time. It has been shown in clinical trials that steroids may diminish the T cell-mediated reduction in transgene expression^9,43,44^, although we recently showed in NHPs that steroids do not prevent the acute reduction in expression observed in rhLDLR-treated animals^45^.

Our hypothesis is that episomal vector genomes are inactivated via intracellular host defense mechanisms that the primate liver has evolved to neutralize the onslaught of viruses from the gut virome^40,41,46^. Insertion of vector genomes into transcriptionally active domains of chromosomal DNA should escape this inactivation. Vector-mediated transgene expression is indeed stable in the livers of lower organisms such as rodents, where host defense pathways are very different^47,48^. Although the data are limited, we believe a similar situation exists in some models of human liver gene therapy. Analysis of liver biopsies of human gene therapy recipients demonstrated the presence of rearranged concatemers, variation in transgene RNA/DNA ratios, and low levels of integrated vector genomes, consistent with our studies in NHPs^20,49^. Indirect measures of transgene expression, such as serum biomarkers, are complicated. A biphasic response to gene therapy was observed using the short half-life biomarker bilirubin in a human CN1 clinical trial, whereas a more uniform kinetic response has been observed in hemophilia A and hemophilia B clinical trials using the longer half-life readout of serum clotting factors^3–5,10^. The durability of AAV transgene expression observed in extrahepatic tissues of all species speaks to the unique behavior of primate liver in modulating AAV transgene expression.

Our findings have implications for improving the efficacy of liver gene therapy. One strategy is to enable the retained episomal AAV genomes located in the discrete nuclear domains to be more transcriptionally active by incorporating *cis* elements, such as different promoters and insulators, or by using drugs that prevent epigenetic silencing^50^. This approach may be effective regardless of whether vector genomes are active and then silenced or are simply never active. Another strategy, based on the proposal that integrated genomes are more likely to confer durable expression, would be to increase the number of vector integrations into safe harbor sites, as is the goal of some editing strategies for treating loss-of-function diseases. In this scenario, we can view editing-based gene insertions as an extension of gene therapy, in which the editing-based insertions are directed to a specific chromosomal location with higher frequency than the broadly distributed background of insertions observed following gene therapy.

## Methods

### AAV vector production

All AAV vectors were produced by the Penn Vector Core at the University of Pennsylvania, as previously described^29^, and visualized using SnapGene (version 6.2.1). Briefly, plasmids expressing rh-β-CG, codon-optimized rhLDLR, codon-optimized hLDLR and enhanced GFP from the *TBG* promoter were packaged within the AAV8 capsid. A vector expressing rh-β-CG from the *TBG* promoter was also packaged with the AAVrh10 capsid. Unique biological materials are available upon request, pending permission by the authors and/or patent holder(s).

### Animal studies

The Institutional Animal Care and Use Committee of the University of Pennsylvania approved all animal procedures in this study. We obtained wild-type adult cynomolgus and rhesus macaques aged 3–6 years (*n* = 20) from Covance Research Products. We conducted NHP studies at the University of Pennsylvania within a facility that is registered with the U.S. Department of Agriculture, accredited by the American Association for Accreditation of Laboratory Animal Care, and assured by the Public Health Service. As previously described^51^, we housed animals in stainless steel squeeze-back cages with perches. All cage sizes and housing conditions were compliant with the Guide for the Care and Use of Laboratory Animals. A 12-h light/12-h dark cycle was maintained and controlled via an Edstrom Watchdog system. Animals were fed Certified Primate Diet 5048 (PMI Feeds) two times per day (morning and evening). We also provided an additional variety of food treats that were fit for human consumption, including fruits, vegetables, nuts and cereals, daily as part of the standard enrichment process. Manipulanda, such as kongs, mirrors, a puzzle feeder and raisin balls, were provided daily. Animals also received visual enrichment and daily human interaction. All interventions were performed during the light cycle, and animals were fasted overnight before being anesthetized.

On study day 0, rhesus macaques received 10^13^ genome copies per kg (body weight) AAV8.TBG.rh-β-CG (*n* = 6), AAVrh10.TBG.rh-β-CG (*n* = 6), AAV8.TBG.rhLDLR (*n* = 2), AAV8.TBG.hLDLR (*n* = 2) or AAV8.TBG.GFP (*n* = 2) as a 10-ml infusion of vector into the saphenous vein at a rate of 1 ml min^–1^ via an infusion pump (Harvard Apparatus). Macaques were anesthetized, and blood was collected on selected days via the femoral vein for analysis of complete blood counts, clinical chemistries and coagulation panels by Antech. We determined neutralizing antibody titers using serum samples taken before the initiation of the study, as previously described^52^. All animals had neutralizing antibody titers of <1/5 for the administered AAV capsid before vector administration. At baseline and throughout the in-life phase of the study, we evaluated the animals for serum biomarkers to determine the transgene expression of either rh-β-CG (as measured by enzyme-linked immunosorbent assay, as previously described^53^) or LDL (as a biomarker for rhLDLR and hLDLR transgene expression). Lipid panel analysis was performed by Antech GLP.

We performed liver biopsies on NHPs throughout the in-life phase of the studies (on study days 14 and 98 for NHPs receiving AAV8.TBG.rh-β-CG or AAVrh10.TBG.rh-β-CG and on study days 14, 77, and 224 for NHPs receiving AAV8.TBG.rhLDLR, AAV8.TBG.hLDLR, or AAV8.TBG.GFP). We conducted a mini-laparotomy procedure to isolate liver tissue and divided the collected samples for histopathology analysis (that is, fixed in 10% neutral buffered formalin) and molecular analysis (that is, frozen on dry ice and stored at −60 °C or colder).

### Necropsy

At day 182 after vector administration for NHPs treated with rh-β-CG vectors and at day 760 for NHPs treated with rhLDLR, hLDLR, and GFP vectors, animals were euthanized and necropsied.

### Vector genome copy and transgene RNA analysis

Tissue samples were snap-frozen at the time of biopsy or necropsy, DNA was extracted using a QIAamp DNA Mini kit (Qiagen), and DNase-treated total RNA was isolated from 100 mg of tissue. RNA was quantified by spectrophotometry and reverse transcribed to cDNA using random primers. We detected and quantified vector genome copy levels in extracted DNA and transgene expression in extracted RNA by qPCR, as previously described^29,51^. Briefly, vector genome copy and RNA levels were quantified using primers and a probe designed for a vector-specific sequence.

### IHC for CG

We performed IHC for CG on formalin-fixed, paraffin-embedded liver sections. Sections were deparaffinized through a xylene and ethanol series, boiled for 6 min in 10 mM citrate buffer (pH 6.0) for antigen retrieval, and treated with 2% H_2_O_2_ (15 min), avidin/biotin-blocking reagents (15 min each; Vector Laboratories) and blocking buffer (1% donkey serum in PBS and 0.2% Triton X-100 for 10 min). We then incubated the sections with rabbit serum to human CG (Abcam, ab9376; diluted 1:200) for 1 h at 37 °C, followed by incubation with biotinylated secondary antibodies (45 min at room temperature; Jackson ImmunoResearch) diluted in blocking buffer according to the manufacturer’s recommendations. We used a Vectastain Elite ABC kit (Vector Laboratories) with 3,3′-diaminobenzidine as a substrate to stain bound antibodies according to the manufacturer’s instructions. No counterstain was applied to the sections to facilitate quantification.

For quantification, we acquired ten random images from each IHC-stained section with a ×10 objective. Using ImageJ software (version 1.52a; http://rsb.info.nih.gov/ij/), we measured the area positive for CG IHC and the area occupied by central and portal veins for each image to calculate the average percentage of CG^+^ area of liver tissue (excluding vein areas).

### ISH for vector DNA and transgene RNA

We performed ISH on formalin-fixed paraffin-embedded liver sections using an RNAscope Multiplex Fluorescent Reagent kit v2 assay (Advanced Cell Diagnostics) following the manufacturer’s protocol. Probes for two-plex ISH were synthesized by Advanced Cell Diagnostics and designed as non-overlapping probe pairs, where one probe is specific for DNA (binding to the antisense strand), and the second probe hybridizes to RNA. Probes for DNA were stained first and detected by Opal 520 precipitates (imaged with a filter set for fluorescein isothiocyanate), and probes binding RNA/DNA were stained in a second step with Opal 570 deposits (imaged with a rhodamine filter set). Reactive Opal dyes were purchased from Akoya Biosciences, and images were acquired with a Nikon Eclipse Ti-E fluorescence microscope. For some sections, we acquired confocal images using a Leica TCS SP5 confocal microscope with an acousto-optical beam splitter.

To quantify ISH^+^ cells, we scanned stained sections with an Aperio Versa fluorescence slide scanner (Leica Biosystems) and analyzed the sections using Visiopharm software (version 2020.06.0.7872) with applications that detect either the probe signal for DNA inside DAPI-stained nuclei or the probe signal for RNA in the cytoplasm.

### ISH with nucleolus localization

After performing ISH, as described above, we further stained some sections with an antibody to fibrillarin as a nucleolar marker. After treatment with 0.5% Triton X-100 in PBS for 2 h and blocking with 1% donkey serum/0.2% Triton X-100 in PBS for 1 h, sections were incubated with rabbit anti-fibrillarin (Abcam, ab166630; diluted 1:100 in 0.5% Triton X-100/PBS) overnight at 4 °C, followed by incubation with Cy5-labeled secondary antibody (donkey anti-rabbit, Jackson ImmunoResearch; diluted 1:100 in 0.5% Triton X-100/PBS for 1 h). We mounted sections using ProLong Gold Antifade Mountant with DAPI (Invitrogen).

### Nuclei isolation and snRNA-seq

To isolate nuclei from frozen tissue samples, a modified version of previously published nuclei isolation procedures was used^54^. For these isolations, all buffers and samples were maintained on ice throughout the procedure to maintain nuclei integrity. Buffers were typically made as described below, cooled in advance and supplemented immediately before use to make ‘complete’ buffers at a final concentration of 1 mM DTT, 0.8 U/µl RNase inhibitor (Protector RNase Inhibitor, Roche), and 1× protease inhibitor (complete mini EDTA-free, Roche). To isolate nuclei, ~25 mg of frozen tissue was minced with a scalpel and transferred to a pre-chilled 2-ml Dounce homogenizer with 1 ml of cold complete lysis buffer (0.32 M sucrose, 5 mM CaCl_2_, 3 mM magnesium acetate, 0.1 mM EDTA, 10 mM Tris-HCl (pH 8.0), and 0.1% Triton X-100). The tissue was homogenized with ten strokes each of pestle A and then B, sequentially passed through prewet 100-µm and 30-µm filters and collected in a sterile tube. The homogenizer and filters were washed with an additional three volumes of complete lysis buffer collected with the filtered sample. Two volumes of sample was then layered on top of one volume of cold complete isolation buffer (1.8 M sucrose, 3 mM magnesium acetate, and 10 mM Tris-HCl (pH 8.0)) and centrifuged at 21,000*g* for 45 min at 4 °C. The supernatant was then carefully removed, and 100 µl of complete resuspension buffer (250 mM sucrose, 25 mM KCl, 5 mM MgCl_2_, and 20 mM Tris-HCl (pH 7.2)) was added to the tube without mixing. Samples were incubated on ice for 10–15 min, and nuclei were fully resuspended by gently pipetting up and down 20–30 times and counted with an automated cell counter (Countess 3, Thermo Fisher). For submission of snRNA-seq samples, the concentration was adjusted to ~1 × 10^6^ nuclei per ml (range of 0.7 × 10^6^–1.2 × 10^6^ nuclei per ml), as per the 10x Genomics sample loading guidelines. To identify single-nuclei transcriptomes, the manufacturer’s protocol was followed to achieve ~10,000 partitioned nuclei per sample using the Chromium Controller and 3′ Gene Expression Assay (version 3, 10x Genomics). Nuclei partitioning, reverse transcription and cleanup, cDNA amplification, and library construction and cleanup were all performed as described in the manufacturer’s protocol, and libraries were sequenced on an Illumina NextSeq2000 to an average depth of ~34,000 reads per nuclei. In addition to liver samples from NHPs described in these studies and to enable analysis of transduction at an early time point, we used samples from two additional animals previously administered AAV8.TBG.GFP and necropsied at day 7 (ref. ^17^).

### snRNA-seq analysis

After sequencing, demultiplexed fastq files were passed through the Cell Ranger count pipeline (version 5.0.1; 10x Genomics) and aligned against a custom reference genome consisting of the rhesus macaque reference (Mmul_10) and the complete annotated plasmid sequence used in the generation of the rAAV vector. Cell Ranger-generated count matrices were then further analyzed within R, with R packages maintained by Bioconductor (version 3.16), using the Seurat package (version 4.3), as previously described^55^. Each individual sample dataset was normalized using the sctransform method, and principal component analysis, UMAP and nuclei clustering were all performed using standard functions within Seurat. For cohort representations, the individual normalized datasets were integrated together by cohort based on the expression of a set of anchor genes using Seurat functions. Cell-type annotation was determined by examining the level of expression of a set of cell-type-specific genes across each cluster of the integrated datasets using the following gene signatures: *TTN*, *TF*, *FGG*, *FGA*, *FGB*, *SERPINA1*, *CPS1*, *CYP3A7* and *CYP2E1* (hepatocytes); *FCGR2A*, *STAB2*, *BMPER*, *NAV1*, *TIE1*, *FCN3*, *LYVE1*, *CLEC4G* and *F8* (LSECs); *CMKLR1*, *HTR7*, *AOAH*, *CPVL*, *MSR1*, *CD163*, *MRC1*, *CD200R1*, *ITGAX*, *ADGRE1* and *FCGR1A* (macrophages); *CMKLR1*, *HTR7*, *AOAH*, *CPVL*, *MSR1*, *CD163*, *MRC1*, *CD200R1*, *TIMD4*, *ADGRE1*, *CLEC4F* and *FCGR1A* (Kupffer cells); *RBMS3*, *PTH1R*, *CCBE1*, *C7*, *ITGA9* and *GRK5* (hepatic stellate cells); *LCK*, *CD247*, *STAT4*, *BCL11B*, *RASGRP1*, *CD3G*, *CD3D* and *CD3E* (T and natural killer cells); *LAMC1*, *LAMC2*, *BEND5*, *VEPH1*, *SLC28A3*, *GRHL2* and *PKHD1* (cholangiocytes) and *FCRL1*, *FCMR*, *BANK1*, *IRF4*, *BLK*, *MS4A1*, *PAX5* and *CD19* (B cells). Data visualizations were completed within Seurat and ggplot2 (version 3.4.0)

### Next-generation sequencing for AAV integration analysis

We identified AAV integration sites in the host genome by ITR-seq, as previously described^18^. Briefly, purified liver DNA was sheared using an ME220 focused ultrasonicator, end repaired, A tailed and ligated to unique Illumina Y adapters that contain a sample barcode and a randomly assigned UMI sequence (UMI in the form of NNWNNWNN). Using AAV ITR and adapter-specific primers, we amplified ITR-containing DNA fragments and generated next-generation sequencing-compatible libraries. We sequenced the DNA on a MiSeq instrument (Illumina) and mapped the obtained reads to the rhesus macaque reference genome and the administered AAV vector genome. We used a custom script to identify AAV integration sites from the mapped reads, which was updated since previously described^18^ to streamline steps, replace outdated programs and allow for the detection of integration site clones.

Mapped reads are labeled with the genomic location of the ITR–genome junction, the adapter–genome junction and the UMI sequence on the adapter. The number of unique genome–AAV junctions was determined for each sample, and this number was normalized to 100 genomes based on input DNA. The number of expansions (clones) for each unique genome–AAV junction was determined by the number of reads at the same unique AAV–genome junction that contain unique adapter–genome junctions and unique UMIs. By requiring both a unique adapter position and a unique UMI for a given ITR integration position, we were able to differentiate between reads originating from cell clones (that is, the same ITR position but different adapter position and different UMI) and PCR duplicates (that is, the same ITR position and same adapter position and/or same UMI) with an enhanced degree of accuracy over previous studies.

The sites were annotated as being within a gene-coding region (genic) or outside of a gene-coding region (intergenic) according to the rhesus macaque Mmul_10 RefSeqGene annotation. Integrations within genic regions were further annotated by the distribution of RNA expression in the human liver (data taken from the Human Genome Atlas; www.proteinatlas.org) and for the presence within genes identified as commonly mutated in HCC (*TERT*, *TP53*, *CTNNB1*, *AXIN1*, *ARID1A*, *BAP1*, *KEAP1*, *RB1* and *NFE2L2*) and within genes indicated in rAAV-associated mouse HCC (*DLK1*, *Tax1bp1*, *Hras*, *Sos1*, *Fgf3* and *MEG8* (the mammalian ortholog of the Rian locus)). Expression levels were determined by nx in the human liver for each annotated gene. The following categories were determined: genes not expressed in liver (1 < nx), genes with low expression in liver (1 ≥ nx < 10), genes with medium expression in liver (10 < nx > 100) and genes with high expression in liver (100 ≥ nx). A random distribution represents 10,000 randomly computationally generated genomic loci in the rhesus macaque genome. The number of AAV integration events in each gene category is presented as fold change over random sequences. A value of 1 would represent no difference from the distribution of random loci.

### Enriched long-read sequencing of liver DNA

Total high-molecular-weight DNA was extracted from liver tissue collected at necropsy from NHPs treated with AAV8.TBG.rhLDLR, AAV8.TBG.hLDLR or AAV8.TBG.GFP (day 760). Biotinylated probes complementary to the ITRs, enhancer, promoter, transgene, woodchuck hepatitis virus post-transcriptional regulatory element (when present) and poly(A) sequences of the vector were hybridized with DNA samples, followed by pulldown with streptavidin-conjugated beads. Enriched DNA was subjected to unbiased linear amplification using the repliG system (Qiagen) and submitted for long-read sequencing using the PacBio Sequel II system and HiFi read technology. To generate these HiFi reads, the pool of enriched DNA was assembled into SMRTbell templates, which contain the double-stranded template DNA to be sequenced with single-stranded hairpin adapters on either end, and subjected to sequencing via CCS with 30 h of sequencing time.

The generated HiFi CCS reads (>99% accuracy and *Q* > 20) were mapped to the rhesus macaque and vector genomes with quantitative and qualitative assessments made using a custom analysis pipeline including BEDtools (version 2.30.0), Samtools (version 1.11), Minimap2 (version 2.24), Cutadapt (version 3.4) and Picard (version 2.26.10), along with visualization of individual reads using the Integrative Genomics Viewer (version 2.16.0). The numbers of CCS reads containing a functional transgene with or without a promoter upstream of the transgene sequence were determined. Reads containing flanking genomic DNA and vector DNA were categorized as integrated. The CIGAR string of these AAV/genomic DNA chimeric reads was used to extract the insertions and deletions within the rhesus genome. Reads containing flanking genomic sequence on both ends of vector sequence were broadly assessed for integrity of the adjacent genomic DNA via NCBI BLAST RefSeq Genome Database for Rhesus Macaques. Reads with at least one flanking genomic sequence were assessed for the presence of ITR sequence and whether the ITR was intact or had a break point. An intact ITR sequence was defined as having a length of 165–173 bp. Break points were assessed, and ITRs from the same long read were compared.

### Long-read analysis of vector and plasmid DNA by Oxford nanopore sequencing

Plasmid DNA used for vector production was linearized by restriction enzyme digestion and sequenced by Oxford Nanopore Technologies long-read sequencing. For AAV vectors, lambda genome was spiked in, and DNase was added to remove potentially contaminating DNA. The capsid was then denatured, and the vector genomes were annealed to create a double-stranded template. Library preparations for plasmid DNA and vector were performed using the Ligation Sequencing gDNA Native Barcoding kit (SQK-NBD112.24) from Oxford Nanopore Technologies. A custom script tested for remaining lambda DNA (to ensure that the DNase treatment worked) and allowed for subsequent mapping of long-read sequences to the plasmid DNA sequence, vector ITR-to-ITR genome and/or *trans*- and helper plasmids used in vector production, *Escherichia coli* and HEK293 cell DNA.

### Statistical analysis

Comparisons between time points were performed for vector genome copy, transgene RNA, DNA ISH and RNA ISH levels using paired *t*-tests in the ‘t.test’ function within the R Program (version 4.1.3). We conducted comparisons between vector genome copies and transgene RNA and quantifications of DNA ISH and IHC using linear mixed-effect modeling and the ‘lme’ function in the ‘nlme’ package for R. A *P* value of <0.05 was considered significant.

### Reporting summary

Further information on research design is available in the Nature Portfolio Reporting Summary linked to this article.

## Online content

Any methods, additional references, Nature Portfolio reporting summaries, source data, extended data, supplementary information, acknowledgements, peer review information; details of author contributions and competing interests; and statements of data and code availability are available at 10.1038/s41587-023-01974-7.

### Supplementary information

Supplementary Information Supplementary Figs. 1–8.

Reporting Summary

Source Data Figs. 1–4 Source data for Figs. 1a–d,k,l, 2a–k, 3e and 4a–c.

## Data Availability

All data discussed in the manuscript are available in the main text or Supplementary Materials. ITR-seq data are available on GitHub (https://github.com/Penn-GTP/ITR-seq2_public, version 2.1.1). Complete clinical pathology data can be obtained upon request.
