# Supplementary material for: Integrated vector genomes may contribute to long-term expression in primate liver after AAV administration
Source: Nat Biotechnol. 2023 Nov 6;42(8):1232–42. doi: 10.1038/s41587-023-01974-7 (PMC11324525; doi:10.1038/s41587-023-01974-7)
Supplement: Supplementary file 1 — Supplementary Figs. 1–8. [file 41587_2023_1974_MOESM1_ESM.pdf]

nature biotechnology

Article

https://doi.org/10.1038/s41587-023-01974-7

Integrated vector genomes may contribute to long-term expression in primate liver after AAV administration

In the format provided by the authors and unedited

Supplementary Data

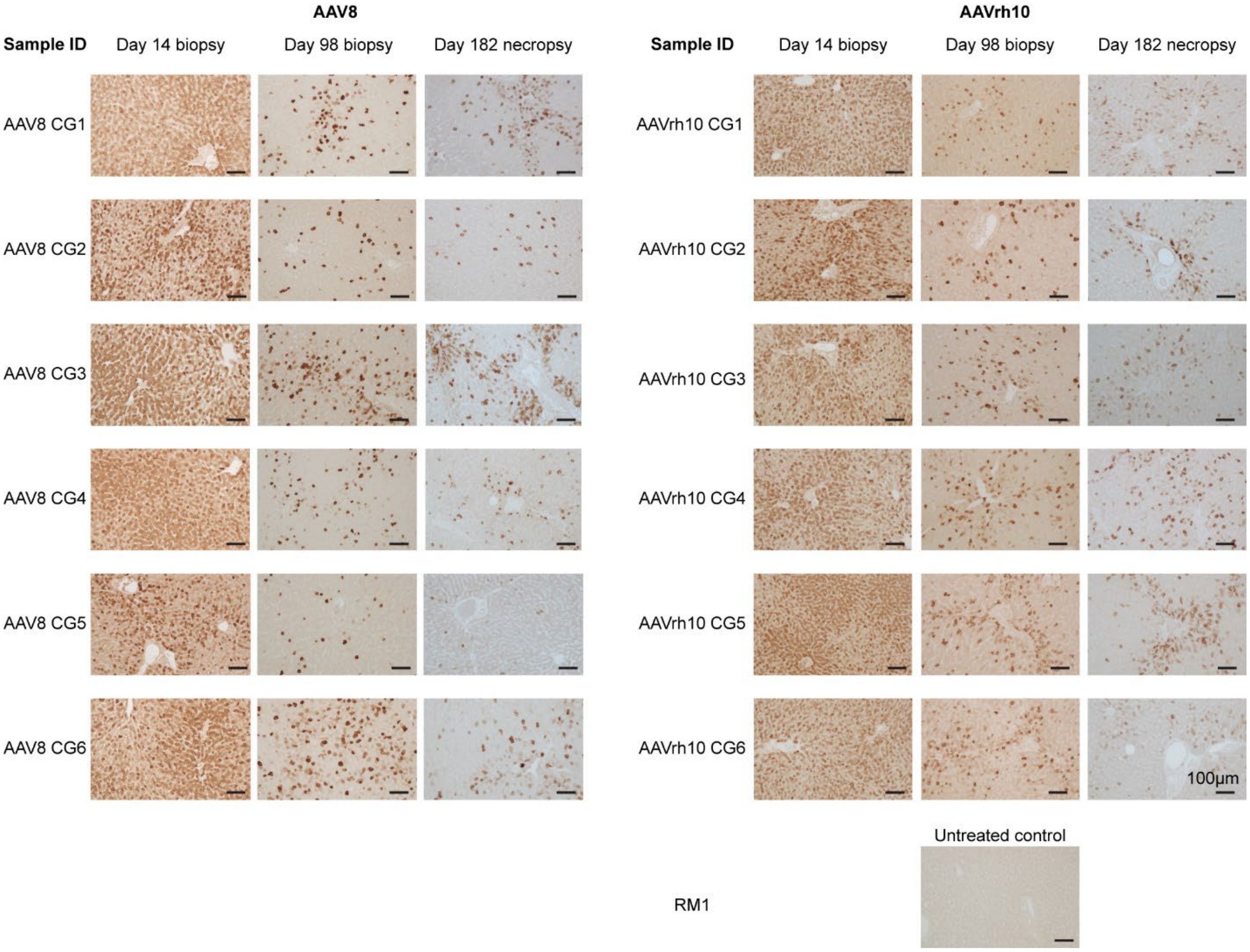

Figure S1. Reduction in self-transgene transduced hepatocytes over time following IV administration of AAV vectors to NHPs. NHPs received IV injections of 10^{13} GC/kg of AAV8 or AAVrh10 vectors expressing the self-transgene rh-β-CG (N=6 per group). Liver tissue was harvested during a liver biopsy procedure (14- or 98-days post-vector administration) or at the time of necropsy (182 days post-vector administration). IHC was performed on liver samples for the CG transgene (brown staining). Images for AAV8 CG1 and AAVrh10 CG1 are in Figure 1 as panels e-j.

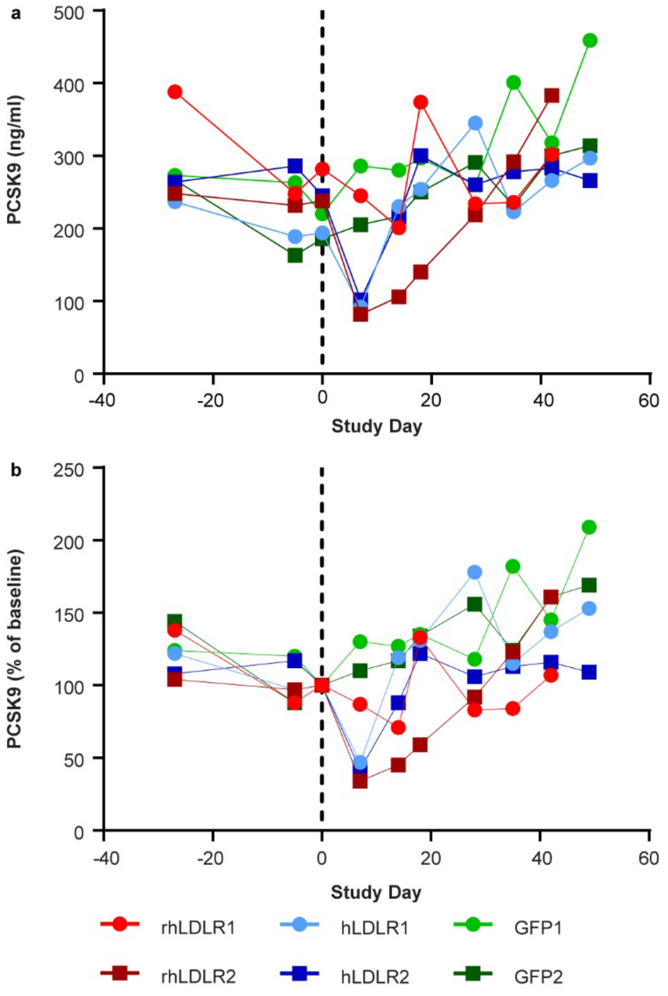

Figure S2. Serum PCSK9 levels following IV administration of AAV8 vectors to NHPs. NHPs received IV injections of 10^{13} GC/kg of AAV vector expressing rhLDLR, hLDLR, or GFP (N = 2/group). Serum PCSK9 levels were evaluated throughout the in-life phase and presented as ng/ml (a) and percent of baseline (day 0) levels (b).

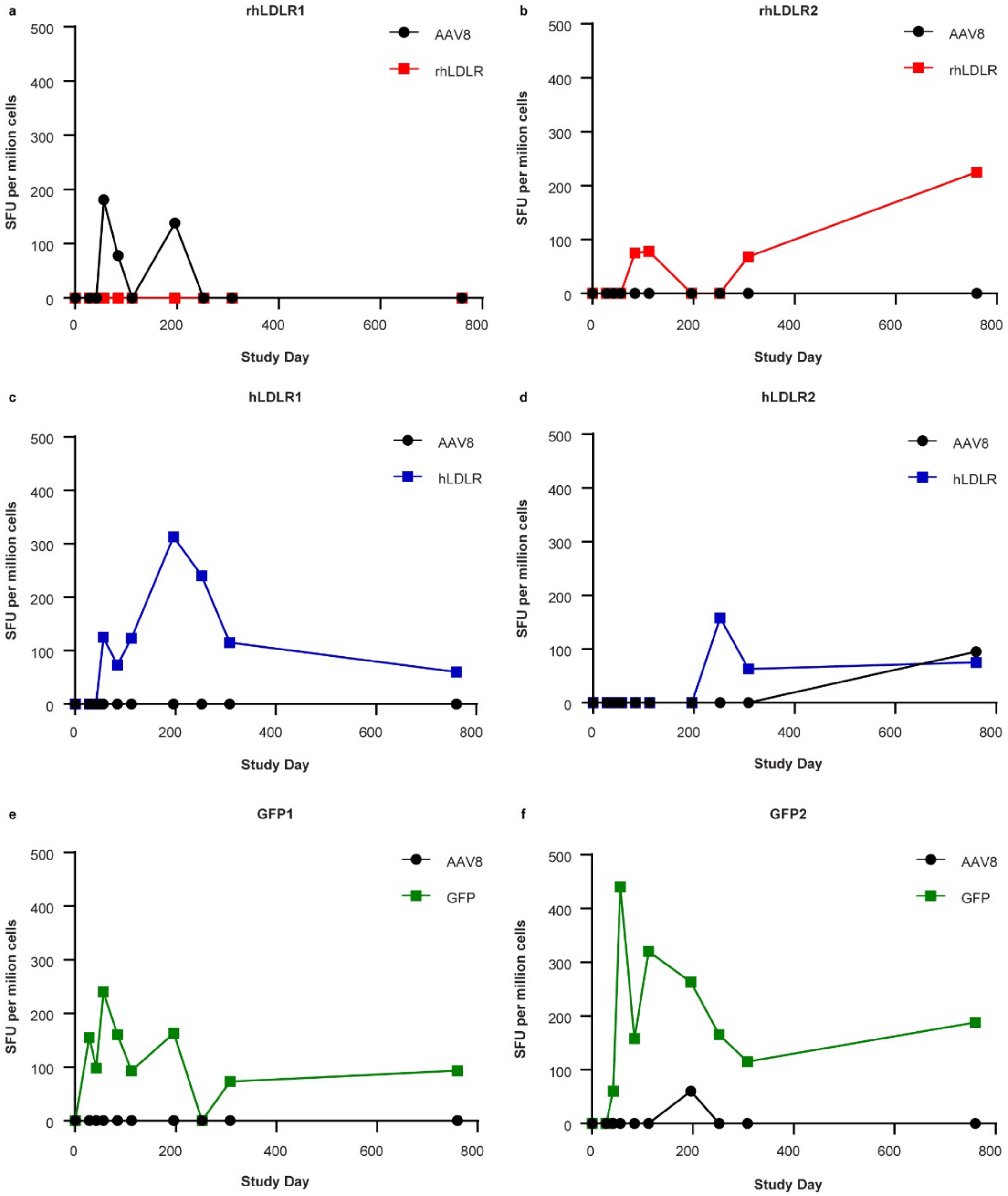

Figure S3. Time course of T-cell immune response following IV administration of vectors expressing self, human, and non-self transgenes in NHPs. NHPs received IV injections of 10^{13} GC/kg of AAV8 vector expressing rhLDLR, hLDLR, or GFP (N = 2/group). Peripheral T-cell responses to the AAV8 capsid and the expressed transgene were measured by IFN-γ ELISPOT throughout the study using peptide libraries specific for the AAV8 capsid (pools A, B, and C) and the transgene (single pool for GFP, pools A-D for rhLDLR and hLDLR). This plot includes only T-cell responses that met the positive criteria, >55 spot forming units (SFU) per 10^6 cells when stimulated with antigen and three times greater than the medium only negative control value (no stimulation).

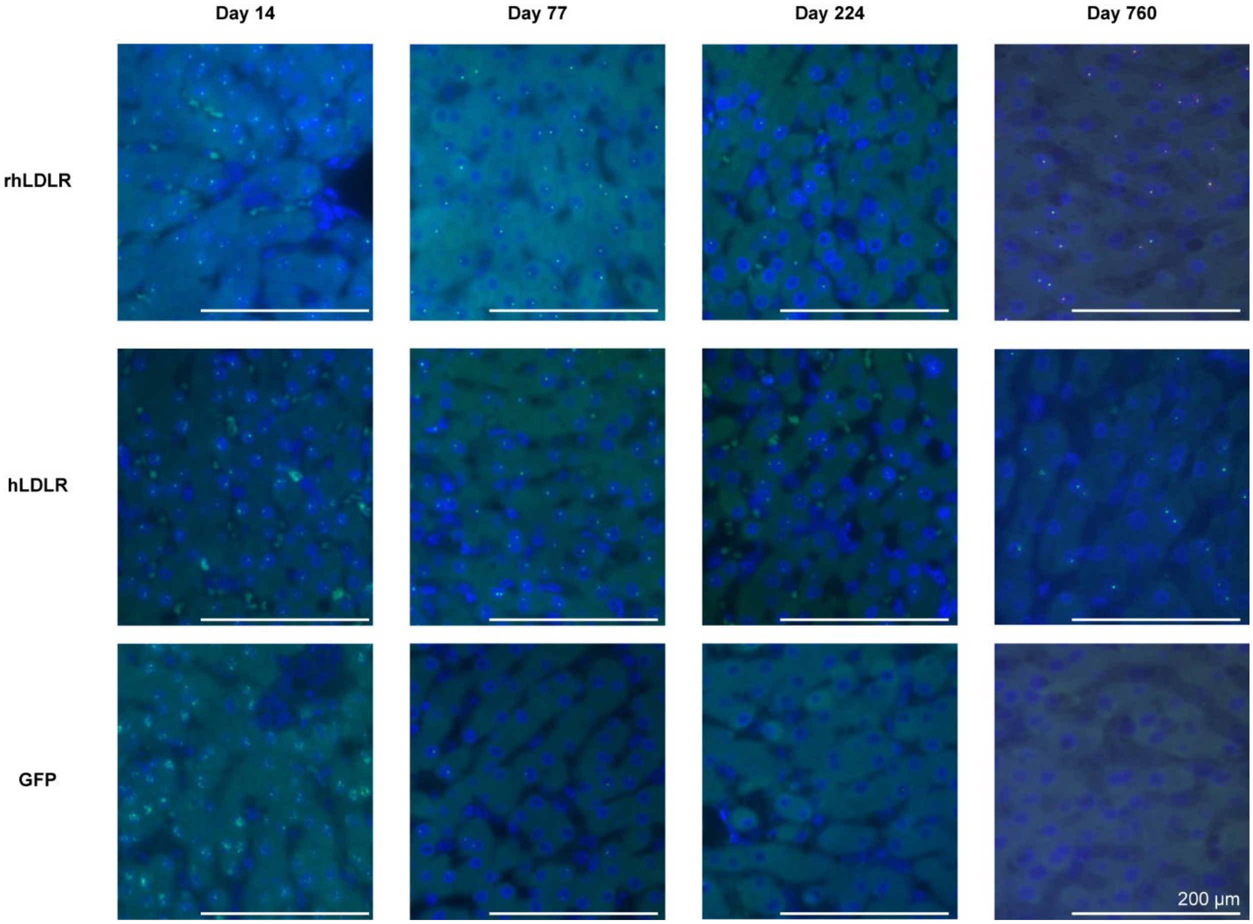

Figure S4. Loss of vector DNA correlates with difference from self following IV administration of AAV vectors. NHPs received IV injections of 10^{13} GC/kg of AAV8 vectors expressing rhLDLR, hLDLR, or GFP (N=2/group). Liver tissue was harvested during a liver biopsy procedure (14-, 77-, and 224-days post-vector administration) or at the time of necropsy (760 days post-vector administration). ISH was performed on liver samples using a DNA-specific probe (binding to the anti-sense strand). Hybridized probes were imaged with a fluorescence microscope. Green, vector DNA; blue, DAPI (nuclear counterstain).

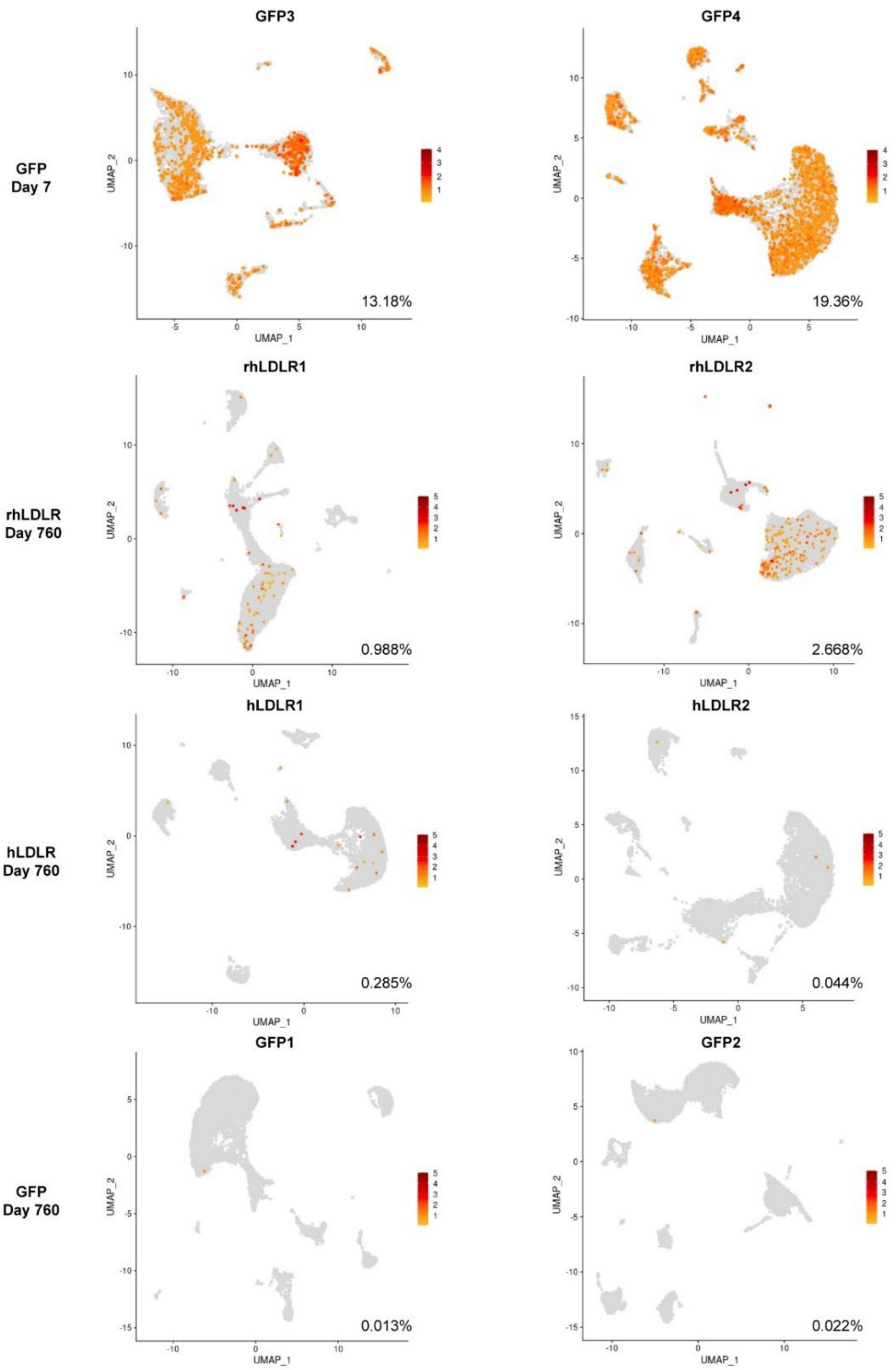

Figure S5. Cellular type and substructure association of transgene RNA following IV administration of AAV vectors. NHPs received IV injections of 7.5×10^{12} GC/kg of AAV8 vector expressing GFP (n=2) or 10^{13} GC/kg of AAV8 vectors expressing rhLDLR, hLDLR, or GFP (N = 2/group). Liver tissue was harvested at necropsy either 7-days or 760-days post-vector administration. Nuclei were extracted and cDNA libraries were created from single nuclei. Nuclei from similar cell types cluster together and the total percentage of nuclei expressing transgene RNA was evaluated.

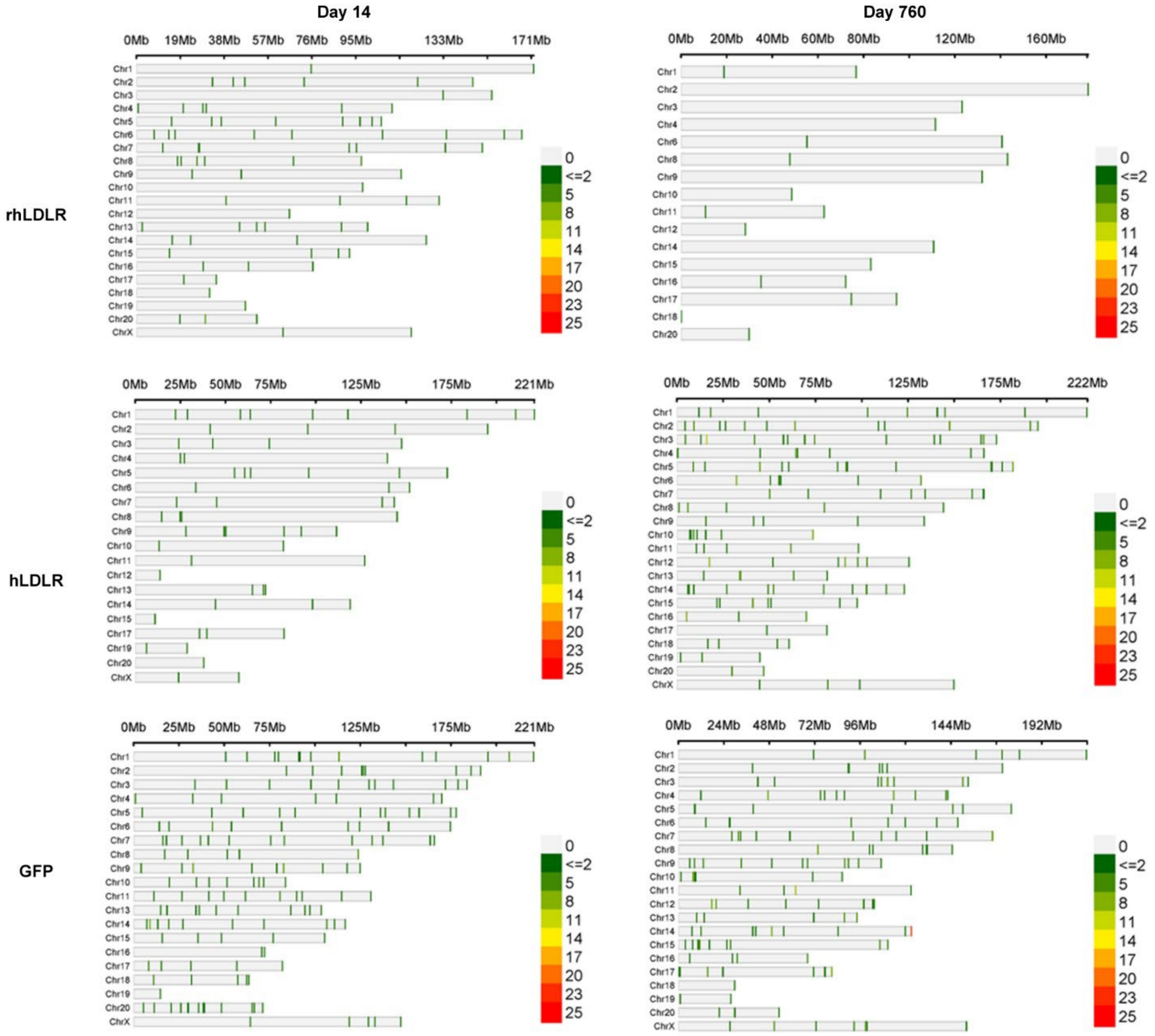

Figure S6. Localization of clonally expanded integrated vector DNA following IV administration of AAV vectors to NHPs. NHPs received IV injections of 10^{13} GC/kg of AAV8 vectors expressing rhLDLR, hLDLR, or GFP (N=2/group). Liver tissue was harvested during a liver biopsy procedure (14-days post-vector administration) or at the time of necropsy the time of necropsy (760-days post-vector administration) and DNA was extracted from liver samples. The number of AAV integration loci in all injected NHPs was determined by ITR-seq and expansion of integration loci was determined by the number of unique molecular identifier and unique genomic positions on the ligated adapters for each ITR-chromosomal junction site. Each line on a chromosome map indicates the location of a detected insertion in which we detected more than 2 or more clones by a single marked line. To be graphed, the insertion site must have been clonally expanded (>1 unique read at the integration loci). The number of expansions at that integration site is indicated by the color of the line per the listed scale. Each individual NHP is represented on an individual chromosome map, grouped by treatment group horizontally (rhLDLR, hLDLR, GFP) and timepoint vertically (Day 14, Day 760).

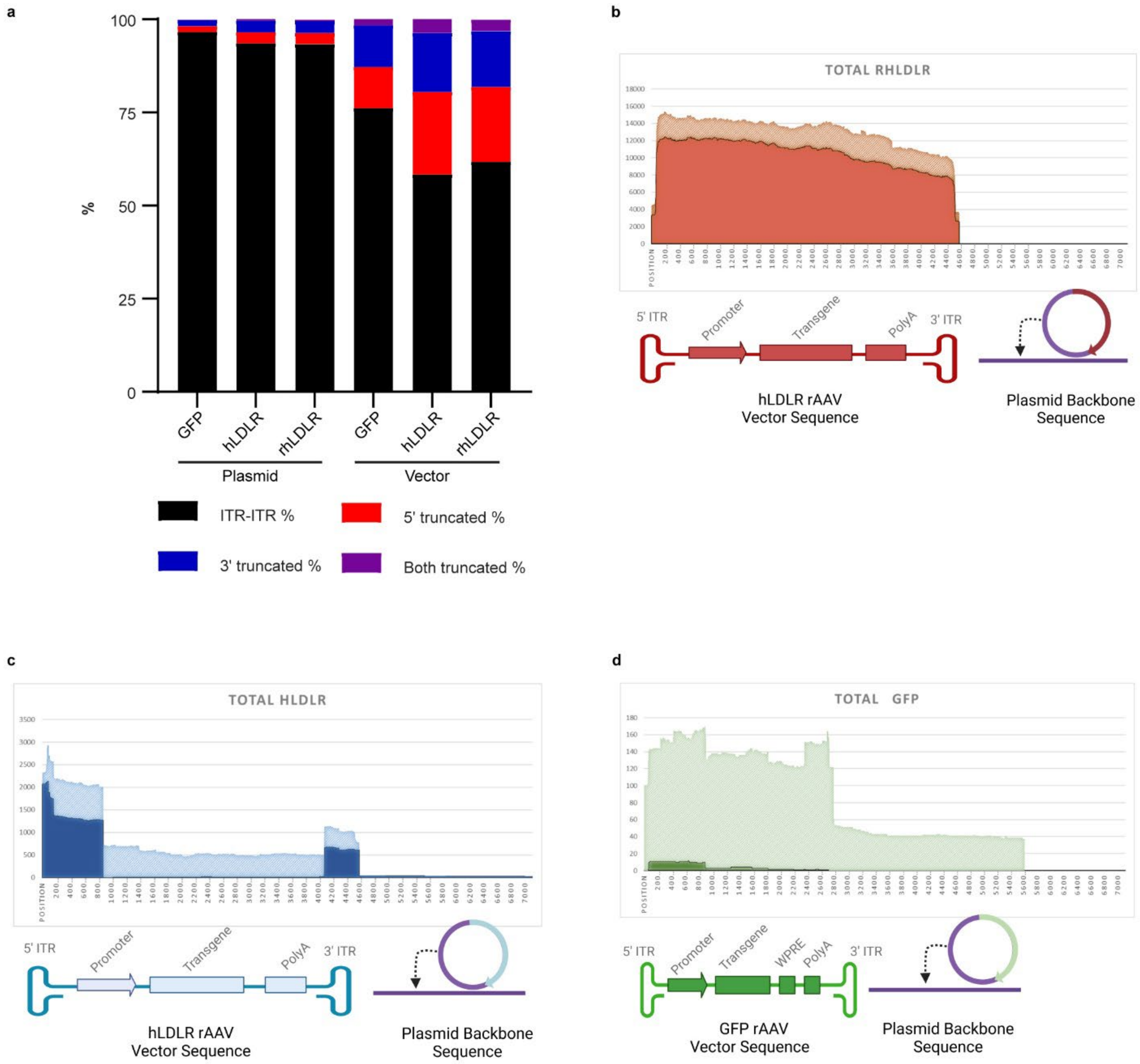

Figure S7. Characterization of vector sequences at a late time point following IV administration of AAV vectors to NHPs. (a) Vectors administered to NHPs and the plasmids used for vector production were linearized by restriction enzyme digestion and sequenced by Oxford Nanopore Technologies long-read sequencing. Reads were then mapped to the plasmid DNA sequence or vector ITR-ITR genome. NHPs received IV injections of 10^{13} GC/kg of AAV8 vectors expressing rhLDLR, hLDLR, or GFP (N=2/group). Liver tissue was harvested during necropsy (760-days post-vector administration). High-molecular-weight DNA was extracted from liver samples and was enriched by hybridization to probes that tiled the vector sequence. HiFi long read sequencing of liver DNA was performed using high consensus accuracy circular consensus sequencing (CCS) on a PacBio Sequel II instrument (>99% accuracy, Q>20) and the total CCS reads from a single run were aligned to input vector (and plasmid) sequence for rhLDLR (b), hLDLR (c), and GFP (d). Light and dark colors indicate each individual NHP in the cohort; red (rhLDLR), blue (hLDLR), and green (GFP).

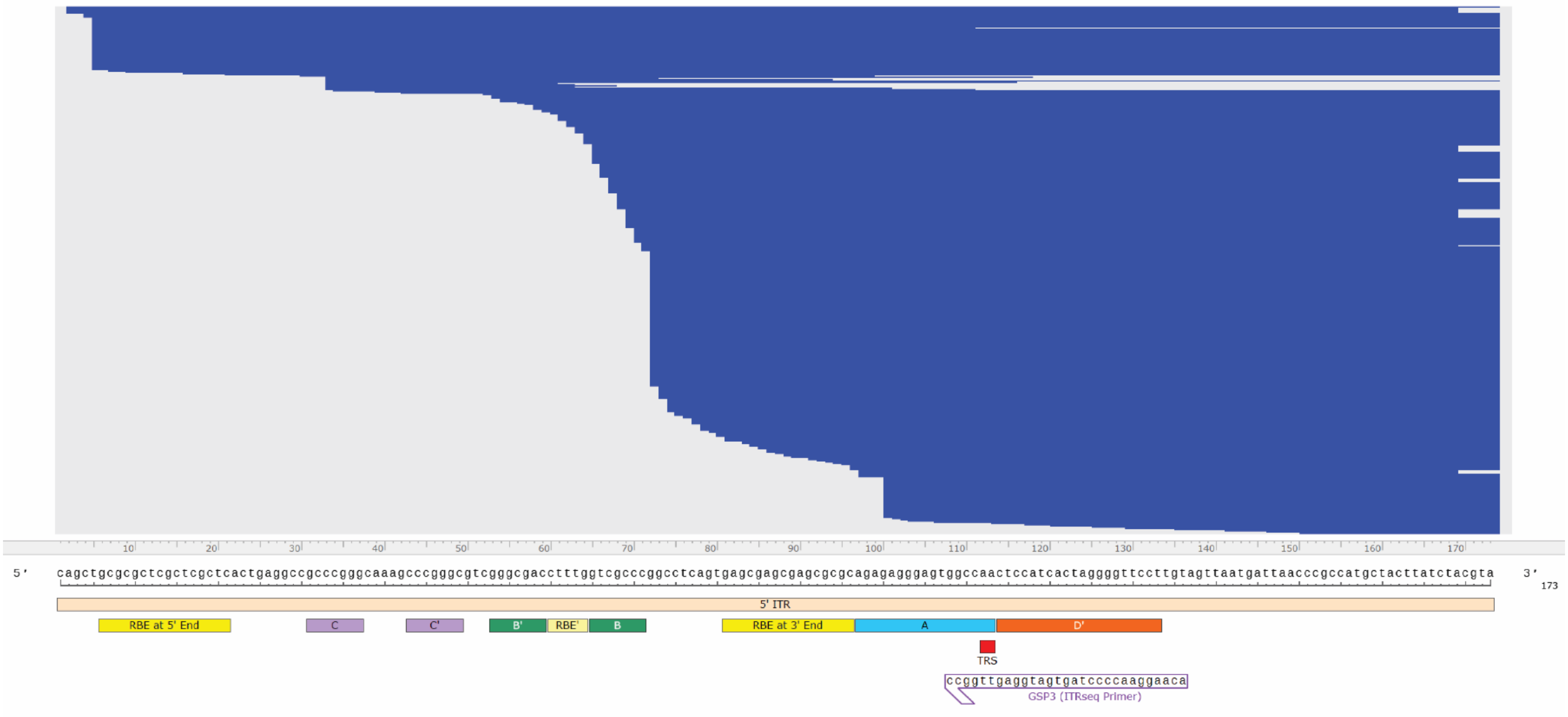

Figure S8. Characterization of the length and breakpoints of ITRs detected within long reads containing vector DNA and flanking genomic sequence. The generated long reads were analyzed on a PacBio Sequel II instrument (>99% accuracy, Q>20). CCS reads were mapped to the rhesus and vector genomes and quantitative and qualitative assessments were made using a custom analysis pipeline and visualization of individual reads using the Integrative Genomics Viewer (IGV). The number of CCS reads containing flanking genomic DNA and vector DNA were assessed for the presence of ITR sequence, and whether the ITR was intact or had a breakpoint. An intact ITR sequence was defined as a length of 165-173bp. Breakpoints and lengths of the ITRs are shown here aligned to the ITR vector sequence used. The GSP3 primer shown represents the primer used in the ITR-seq short read sequencing. Blue lines represent individual ITRs.
