# Supplementary material for: Integrated vector genomes may contribute to long-term expression in primate liver after AAV administration
Source: Nat Biotechnol. 2023 Nov 6;42(8):1232–42. doi: 10.1038/s41587-023-01974-7 (PMC11324525; doi:10.1038/s41587-023-01974-7)
Supplement: Supplementary file 2 — Reporting Summary [file 41587_2023_1974_MOESM2_ESM.pdf]

nature portfolio

Corresponding author(s): James M. Wilson
Last updated by author(s): Jun 27, 2023

Reporting Summary

Nature Portfolio wishes to improve the reproducibility of the work that we publish. This form provides structure for consistency and transparency in reporting. For further information on Nature Portfolio policies, see our Editorial Policies and the Editorial Policy Checklist.

Statistics

For all statistical analyses, confirm that the following items are present in the figure legend, table legend, main text, or Methods section.

n/a	Confirmed	
☐	☒	The exact sample size (*n*) for each experimental group/condition, given as a discrete number and unit of measurement
☐	☒	A statement on whether measurements were taken from distinct samples or whether the same sample was measured repeatedly
☐	☒	The statistical test(s) used AND whether they are one- or two-sided<br>*Only common tests should be described solely by name; describe more complex techniques in the Methods section.*
☐	☒	A description of all covariates tested
☐	☒	A description of any assumptions or corrections, such as tests of normality and adjustment for multiple comparisons
☐	☒	A full description of the statistical parameters including central tendency (e.g. means) or other basic estimates (e.g. regression coefficient) AND variation (e.g. standard deviation) or associated estimates of uncertainty (e.g. confidence intervals)
☐	☒	For null hypothesis testing, the test statistic (e.g. *F*, *t*, *r*) with confidence intervals, effect sizes, degrees of freedom and *P* value noted<br>*Give P values as exact values whenever suitable.*
☒	☐	For Bayesian analysis, information on the choice of priors and Markov chain Monte Carlo settings
☒	☐	For hierarchical and complex designs, identification of the appropriate level for tests and full reporting of outcomes
☒	☐	Estimates of effect sizes (e.g. Cohen's *d*, Pearson's *r*), indicating how they were calculated

Our web collection on statistics for biologists contains articles on many of the points above.

Software and code

Policy information about availability of computer code

Data collection

All data documenting experimental details, study procedures, observations were captured recorded by hand and/or extracted from data reports and maintained by GTP personnel in dedicated notebooks and study binders.

Data analysis

See methods for full details.

ITR-Seq data are available on GitHub (ITR-Seq on GitHub (https://github.com/Penn-GTP/ITR_Seq2, version 2.1.1).

All adeno-associated virus (AAV) vectors were visualized using SnapGene (version 6.2.1).

Using ImageJ software (version 1.52a; Rasband, W. S., National Institutes of Health, USA; http://rsb.info.nih.gov/ij/), we measured the area positive for CG IHC and the area occupied by central and portal veins for each image, which was then used to calculate the average percentage of CG-positive area of liver tissue while excluding vein areas.

To quantify ISH-positive cells, we scanned stained sections with an Aperio Versa fluorescence slide scanner (Leica Biosystems) and analyzed the sections using Visiopharm software (version 2020.06.0.7872; Hoersholm, Denmark) with apps that detect either the probe signal for DNA inside 4′,6-diamidino-2-phenylindole (DAPI)-stained nuclei or the probe signal for RNA in the cytoplasm.

After sequencing, demultiplexed fastq files were passed through the Cell Ranger count pipeline (version 5.0.1; 10x Genomics) and aligned against a custom reference genome consisting of the rhesus macaque reference (Mmul 10) and the complete annotated plasmid sequence used in the generation of the rAAV vector. Cell Ranger-generated count matrices were then further analyzed within R, with R packages maintained by Bioconductor (version 3.16), using the Seurat package (version 4.3). Each individual sample dataset was normalized using the

sctransform method, and principal component analysis (PCA), uniform manifold approximation and projection (UMAP), and nuclei clustering were all performed using standard functions within Seurat. For cohort representations, the individual normalized datasets were integrated together by cohort based on the expression of a set of anchor genes using Seurat functions. Data visualizations were completed within Seurat and ggplot2 (version 3.4.0).

The generated HiFi CCS reads (>99% accuracy, Q>20) were mapped to the rhesus and vector genomes with quantitative and qualitative assessments made using a custom analysis pipeline including BEDtools (version 2.30.0), Samtools (version 1.11), Minimap2 (version 2.24), Cutadapt (version 3.4), and Picard (version 2.26.10), along with visualization of individual reads using the Integrative Genomics Viewer (IGV; version 2.16.0).

Comparisons between time points were performed for vector GC, transgene RNA, DNA ISH, and RNA ISH levels using paired t-tests in the "t.test" function within the R Program (version 4.1.3). We conducted comparisons between vector GCs and transgene RNA and quantifications of DNA ISH and IHC using linear mixed-effect modeling using the "lme" function in the "nlme" package for R.

For manuscripts utilizing custom algorithms or software that are central to the research but not yet described in published literature, software must be made available to editors and reviewers. We strongly encourage code deposition in a community repository (e.g. GitHub). See the Nature Portfolio guidelines for submitting code & software for further information.

Data

Policy information about availability of data

All manuscripts must include a data availability statement. This statement should provide the following information, where applicable:

- Accession codes, unique identifiers, or web links for publicly available datasets
- A description of any restrictions on data availability
- For clinical datasets or third party data, please ensure that the statement adheres to our policy

All data discussed in the manuscript are available in the main text or supplementary materials (see extended data file 1). Complete clinical pathology data can be obtained upon request.

Human research participants

Policy information about studies involving human research participants and Sex and Gender in Research.

Reporting on sex and gender

Use the terms sex (biological attribute) and gender (shaped by social and cultural circumstances) carefully in order to avoid confusing both terms. Indicate if findings apply to only one sex or gender; describe whether sex and gender were considered in study design whether sex and/or gender was determined based on self-reporting or assigned and methods used. Provide in the source data disaggregated sex and gender data where this information has been collected, and consent has been obtained for sharing of individual-level data; provide overall numbers in this Reporting Summary. Please state if this information has not been collected. Report sex- and gender-based analyses where performed, justify reasons for lack of sex- and gender-based analysis.

Population characteristics

Describe the covariate-relevant population characteristics of the human research participants (e.g. age, genotypic information, past and current diagnosis and treatment categories). If you filled out the behavioural & social sciences study design questions and have nothing to add here, write "See above."

Recruitment

Describe how participants were recruited. Outline any potential self-selection bias or other biases that may be present and how these are likely to impact results.

Ethics oversight

Identify the organization(s) that approved the study protocol.

Note that full information on the approval of the study protocol must also be provided in the manuscript.

Field-specific reporting

Please select the one below that is the best fit for your research. If you are not sure, read the appropriate sections before making your selection.

☒ Life sciences ☐ Behavioural & social sciences ☐ Ecological, evolutionary & environmental sciences

For a reference copy of the document with all sections, see nature.com/documents/nr-reporting-summary-flat.pdf

Life sciences study design

All studies must disclose on these points even when the disclosure is negative.

Sample size

No sample-size calculation was performed, NHPs are a highly representative, translational model that responds well to AAV gene therapy intervention, so minimal numbers were required to detect the reproducible, significant effect

Data exclusions

No data were excluded from the analyses.

Replication	No measures taken to verify reproducibility of experimental findings.
Randomization	Randomization was not relevant to the study, as the investigation focused on the general effects of AAV gene therapy in a wild type population.
Blinding	No blinding was included as analysis was performed in a transgene-specific manner, plus a variety of time points were evaluated when data became available (instead of analysis at the end of the study)

Reporting for specific materials, systems and methods

We require information from authors about some types of materials, experimental systems and methods used in many studies. Here, indicate whether each material, system or method listed is relevant to your study. If you are not sure if a list item applies to your research, read the appropriate section before selecting a response.

Materials & experimental systems

n/a	Involved in the study	
☐	☒	Antibodies
☒	☐	Eukaryotic cell lines
☒	☐	Palaeontology and archaeology
☐	☒	Animals and other organisms
☒	☐	Clinical data
☒	☐	Dual use research of concern

Methods

n/a	Involved in the study	
☒	☐	ChIP-seq
☒	☐	Flow cytometry
☒	☐	MRI-based neuroimaging

Antibodies

Antibodies used	Rabbit serum against human CG (Abcam ab9376), CG antibody is polyclonal (no clone name), Lot# GR153293-7;<br>Rabbit anti-fibrillarin antibody (Abcam ab166630), clone EPR10823(B), Lot# GR118191-19
Validation	rabbit serum against human CG (Abcam ab9376); https://www.abcam.com/hcg-antibody-ab9376.html;<br>rabbit anti-fibrillarin antibody (Abcam ab166630); https://www.abcam.com/fibrillarin-antibody-epr10823b-nucleolar-marker-ab166630.html

Animals and other research organisms

Policy information about studies involving animals; ARRIVE guidelines recommended for reporting animal research, and Sex and Gender in Research

Laboratory animals	Rhesus macaques, male and female, 3 – 6 years old
Wild animals	The study did not involve wild animals.
Reporting on sex	Male and female animals were included in this study but insufficient numbers were collected to permit sex-based analyses.
Field-collected samples	The study did not involve samples collected from the field.
Ethics oversight	The Institutional Animal Care and Use Committee of the University of Pennsylvania approved all animal procedures in this study. We conducted nonhuman primate (NHP) studies at the University of Pennsylvania within a facility that is registered with the United States Department of Agriculture, accredited by the American Association for Accreditation of Laboratory Animal Care, and assured by the Public Health Service.

Note that full information on the approval of the study protocol must also be provided in the manuscript.
